# GenomicSuperSignature facilitates interpretation of RNA-seq experiments through robust, efficient comparison to public databases

**DOI:** 10.1038/s41467-022-31411-3

**Published:** 2022-06-27

**Authors:** Sehyun Oh, Ludwig Geistlinger, Marcel Ramos, Daniel Blankenberg, Marius van den Beek, Jaclyn N. Taroni, Vincent J. Carey, Casey S. Greene, Levi Waldron, Sean Davis

**Affiliations:** 1 Graduate School of Public Health and Health Policy and Institute for Implementation Sciences in Public Health, City University of New York, New York, NY USA; 2 Center for Computational Biomedicine, Harvard Medical School, Boston, MA USA; 3 Genomic Medicine Institute, Lerner Research Institute, Cleveland Clinic, Cleveland, OH USA; 4 Department of Molecular Medicine, Cleveland Clinic Lerner College of Medicine, Case Western Reserve University, Cleveland, OH USA; 5 The Pennsylvania State University, State College, PA USA; 6 Childhood Cancer Data Lab, Alex’s Lemonade Stand Foundation, Bala Cynwyd, PA USA; 7 Channing Division of Network Medicine, Mass General Brigham, Harvard Medical School, Boston, MA USA; 8 Center for Health AI, University of Colorado Anschutz School of Medicine, Denver, CO USA

**Keywords:** Data mining, Data integration, Medical research, Transcriptomics, Machine learning

## Abstract

Millions of transcriptomic profiles have been deposited in public archives, yet remain underused for the interpretation of new experiments. We present a method for interpreting new transcriptomic datasets through instant comparison to public datasets without high-performance computing requirements. We apply Principal Component Analysis on 536 studies comprising 44,890 human RNA sequencing profiles and aggregate sufficiently similar loading vectors to form Replicable Axes of Variation (RAV). RAVs are annotated with metadata of originating studies and by gene set enrichment analysis. Functionality to associate new datasets with RAVs, extract interpretable annotations, and provide intuitive visualization are implemented as the GenomicSuperSignature R/Bioconductor package. We demonstrate the efficient and coherent database search, robustness to batch effects and heterogeneous training data, and transfer learning capacity of our method using TCGA and rare diseases datasets. GenomicSuperSignature aids in analyzing new gene expression data in the context of existing databases using minimal computing resources.

## Abstract (web summary)

Many transcriptomic profiles have been deposited in public archives but are underused for the interpretation of experiments. Here the authors report GenomicSuperSignature for interpreting new transcriptomic datasets through comparison to public archives, without high-performance computing requirements.

## Introduction

The computational infrastructure and skills currently required to leverage the vast quantities of publicly available transcriptomic data render such analyses infeasible for most basic, translational, and clinical researchers. Those who wish to do so must often turn to well-funded computational collaborators with access to significant compute resources in order to provide context and aid in interpreting new experiments. Yet, as public data resources grow, there is a critical need to reduce computational burdens to their application while increasing the breadth of data resources being integrated and analyzed.

Dimensionality reduction has been broadly adopted to transform large transcriptomes onto a smaller number of latent variables representing co-expressed transcripts. Many dimensionality reduction approaches, differing in the optimization and constraint criteria, are available^1^ and there have been multiple attempts to detect biological and technical signals through these lower-dimensional, latent variable representations. Gene co-expression can result from shared function or regulation^2^, association with tissue composition or cell type^3^, and technical batch effects^4^. In the confluence of these factors, dimensionality reduction can assist interpretability and reduce the burden of multiple hypothesis testing, but can also lead to incomplete or misleading interpretation. The valid interpretation would be improved by comparison of latent variables in new datasets to those also present in public transcriptome databases.

Classic methods of dimensionality reduction such as Principal Component Analysis (PCA) and Non-negative Matrix Factorization (NMF) remain widely used in their original form and as bases for newer methods. For example, Single Cell Coordinated Gene Activity in Pattern Sets (scCoGAPS) is an NMF method optimized for large, sparse single-cell RNA sequencing datasets^5^. scCoGAPS recovers features in a source dataset and then projects a new dataset onto this learned latent space through projectR^5,6^. This approach requires users to train their own model and mostly focuses on single-cell RNA sequencing datasets with similar biology. Pathway-Level Information Extractor (PLIER) aims to extract biologically meaningful and interpretable signatures from high dimensional molecular data by identifying latent variables that map to a single gene set or a group of highly related gene sets with positive correlations^7^. MultiPLIER applies the PLIER approach to transfer learned patterns from a large public dataset to rare diseases^8^. Other tools focus on recovering consistent signals from multiple datasets across distinct platforms^9,10^, increasing interpretability^11^, simple database search^12^, or transfer learning between datasets of a specific type^13,14^. However, none of these tools enable a routine exploratory analysis of new studies through comparison to large public transcriptome databases (Supplementary Note 1). Also, these tools do not provide a reference catalog for transfer learning from large public databases, or in the case of MultiPLIER, require substantial computing resources and bioinformatics expertise.

Here, we introduce GenomicSuperSignature, a toolkit for interpreting new RNA-seq datasets in the context of a large-scale database of previously published and annotated results. As an exploratory data analysis tool, GenomicSuperSignature matches PCA axes in a new dataset to an annotated index of Replicable Axes of Variation (RAV) that are represented in previously published independent datasets. GenomicSuperSignature also can be used as a tool for transfer learning^15^, utilizing RAVs as well-defined and replicable latent variables defined by multiple previous studies in place of de novo latent variables. The interpretability of RAVs is enhanced through annotations by MEdical Subject Headings (MeSH) and Gene Set Enrichment Analysis (GSEA). Through the use of pre-built, pre-annotated, dimension-reduced RAVs, GenomicSuperSignature leverages knowledge from tens of thousands of samples and from PubMed and MSigDB^16^, to the dataset at hand within seconds on an ordinary laptop. We demonstrate these functionalities in colorectal carcinoma, breast invasive carcinoma, systemic lupus erythematosus, and rare inflammatory disease. GenomicSuperSignature is implemented as an R/Bioconductor package for straightforward incorporation into popular RNA-seq analysis pipelines.

## Results

**Fig. 1:**
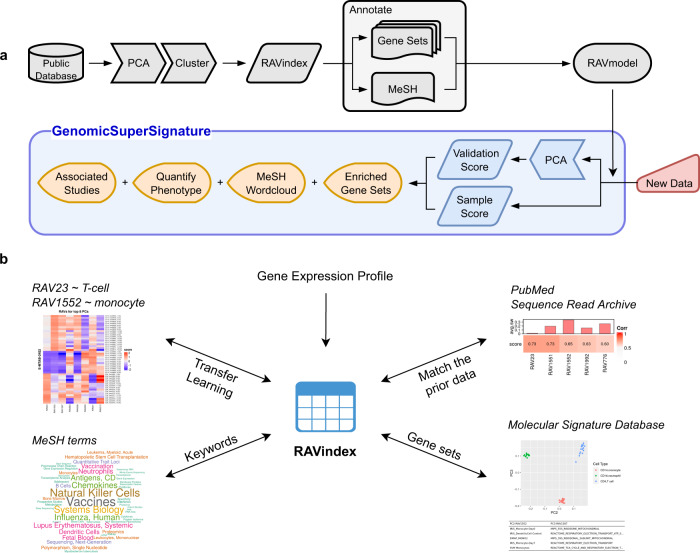
Overview of GenomicSuperSignature. **a** Schematic illustration of RAVmodel construction and GenomicSuperSignature application. Building the RAVmodel (gray) was performed over several days on 24 cores with 128 Gb memory. Users can apply RAVmodel on their data (red) using the GenomicSuperSignature R/Bioconductor package (blue), which operates on a time scale of seconds for exploratory data analysis (orange) on a typical laptop computer. **b** Information assembled as RAVmodel, a single R object. RAVmodel connects different public databases and prior information through RAVindex (Supplementary Fig. 1b), creating the interconnected resources illustrated here. Through GenomicSuperSignature R/Bioconductor package and the accompanying RAVmodel, users can instantly access and explore the diverse public databases from multiple entry points such as gene expression profiles, publications, study metadata, keywords in MeSH terms and gene sets.

The current RAVmodel is trained on 536 studies containing 44,890 human RNA sequencing profiles. This RAVmodel is associated with 18,798 (4,430 unique) MeSH terms and 70,687 (1,784 unique) MSigDB curated (C2) gene sets. This integration of data resources (Fig. 1b) is accompanied by tools in the GenomicSuperSignature R/Bioconductor package for the interpretation of new datasets (Fig. 1a, Supplementary Fig. 1c). We demonstrate this application of public data in three examples. First, using TCGA datasets, we show that new data can be rapidly associated with related studies, gene sets, and MeSH terms. Second, we show that the RAVmodel trained from diverse RNA-seq experiments identified colon cancer transcriptome subtypes more closely associated with clinicopathological variables than the subtypes previously identified by a meta-analysis of a focused colorectal carcinoma (CRC) microarray compendium. Lastly, we show that neutrophil counts of two independent datasets can be interpreted and inferred through a single RAV, providing a quantitative measure of neutrophil count from transcriptome data. These examples, along with sensitivity analyses and simulations, demonstrate that the RAVmodel and associated GenomicSuperSignature software (see Supplementary Note 2 for implementation details) constitute robust, general-purpose methods for the interpretation of transcriptome data.

### Sensitivity analysis and simulation

Model training methods were optimized for robustness, simplicity, computational cost, and validity (see Methods). Briefly, the RAVmodel was trained on the RNA-seq Sample Compendia of refine.bio^17^. We analyzed TPM count data using PCA following log-transformation, then identified clusters of similar principal components (PCs) from independent datasets using hierarchical clustering on Spearman distance and ward.D agglomeration. This approach was compared to alternatives based on (1) ability to group synthetic true-positive PCs, (2) separation of synthetic true-negative PCs added to training data, (3) magnitude of changes in the results compared to the simplest method, and (4) maintenance of RAVs identified from a focused training dataset when adding unrelated datasets. Alternative approaches considered but not selected for training the final model included NMF, Independent Component Analysis (ICA), PLIER^7^ and MultiPLIER^8^, Variance-Stabilizing Transformation (VST)^18^, combining training datasets into a single dataset instead of analyzing them independently, increasing the number of PCs included per dataset, and alternative clustering algorithms including graph-based clustering. These assessments are described in the “Build RAVmodels” section of the Methods.

### The RAVmodel

A RAVmodel is composed of an index of RAVs (RAVindex), model metadata, and annotation modules linked through RAVs (Supplementary Fig. 1b). As detailed in Methods, each of 536 studies were subjected to PCA and the resulting 10,720 PCs were then clustered to produce RAVs, a vector containing the average of similar loadings collected from distinct studies. The current RAVindex has 4764 RAVs and 1378 are ‘single-element’ clusters (ie., a cluster with only one PC). By definition a single-element cluster is not a ‘repetitive’ signal, leaving only 3386 RAVs, though we will continue to refer to all clustered PCs, including single-element clusters as RAVs. Thus, we compressed the information from 44,890 samples into 3386 RAVs, which is less than 1/10 of the initial number of samples. Also, 417 out of 536 training datasets have 40,746 genes and the other 119 training datasets have 41,255 genes, while the RAVindex uses only 13,934 common genes among the top 90% varying genes of all samples. Thus, our method achieves an efficient data compression, maintaining significant information in ~3% of the initial volume of the training data.

The distribution of the number of PCs in RAVs shows that most RAVs consist of a small number of clustered PCs (Supplementary Fig. 4). When we exclude single-element clusters, about 65% of RAVs (2212 out of 3386) are composed of two PCs. The mean cluster size is 2.759 PCs per RAV with the largest cluster containing 24 PCs. Interestingly, the proportion of variance explained by PCs varies systematically with the number of PCs in the RAVs. The majority of PCs in one- and two-element RAVs, on average, explain a relatively low proportion of variance with an increasing proportion of PCs explaining more variance as RAV cluster size increases (Supplementary Data 3). This suggests that RAVs from small clusters tend to represent weak and less common signals. We chose to propagate the ‘single-element’ RAVs into our final models for two reasons: (1) If any new data is validated by those ‘single-element’ RAVs, they become ‘repetitive’ signals and thus, could lead to new hypotheses and (2) by keeping all RAVs, we include all potential PCs in the RAVmodel and support different use cases. Since metadata associated with all RAVs are readily accessible, end users can filter downstream results based on cluster sizes or other RAV properties.

We assess the number of enriched gene sets for each RAV from ‘RAVmodel_C2’ annotated with MSigDB C2 gene sets and ‘RAVmodel_PLIERpriors’ annotated with three gene sets provided through PLIER package (see Methods). About 40% of RAVs in RAVmodel_C2 and 50% of RAVs in RAVmodel_PLIERpriors do not have any enriched pathway and the majority of them are one- or two- element clusters (Supplementary Fig. 5), suggesting that the smaller clusters are less likely to represent biological features. Because there are RAVs annotated with only one input annotation, MSigDB C2 or PLIERpriors, we include all the RAVs to make our model cover diverse annotation databases. We further evaluate the scope of biological features represented by RAVmodel through two model validation measures, pathway coverage and pathway separation, used to evaluate MultiPLIER model^8^. Pathway coverage is defined as the proportion of pathways annotating RAVs out of all the gene set terms provided. Pathway coverage of RAVmodel_C2 is 0.32. The recount2_MultiPLIER has the pathway coverage of 0.42 while the RAVmodel_PLIERpriors which uses the same gene set as recount2_MultiPLIER has 0.64 pathway coverage. Pathway separation is defined as the ability of the model to keep non-overlapping signatures that can differentiate biologically similar pathways. Three biological subjects were tested on RAVmodel_PLIERpriors - type I versus type II interferon, neutrophil versus monocyte, and G1 versus G2 cell cycle phases. RAVmodel_PLIERpriors can successfully separate them either with the top one or the top five enriched pathways.

Redundancy within the cluster is defined as the cluster containing more than one PC from the same study. The majority of RAVs (78%, 2628 out of 3386 non-single-element RAVs) consist of PCs from unique studies. 622 non-single-element RAVs are composed of only one study and 80% of them have no or only one MSigDB C2 pathway enriched.

To guide the interpretation, GenomicSuperSignature gives a message when the output includes any of the following RAVs: (1) single-element RAVs, (2) RAVs with no or too-many enriched pathways, where ‘too-many’ is defined as 5% of input gene sets (276 and 31 for MSigDB C2 and PLIERpriors, respectively), (3) non-single-element RAVs constructed from a single study. These criteria together include 2557 RAVs.

### Connecting new data with the existing databases

**Fig. 2:**
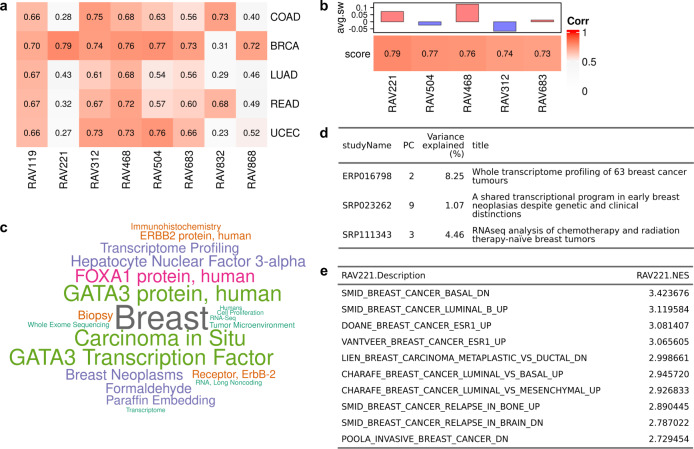
Connecting new datasets to existing databases. **a** GenomicSuperSignature provides a rich resource for understanding new or user-supplied datasets in the context of existing datasets summarized in the RAVmodel. **a** Validation of multiple TCGA RNA-seq datasets. Each dataset was subjected to PCA and Pearson correlation coefficients between top PCs and all possible RAVs were calculated. RAVs with Pearson coefficients above 0.7 in at least one dataset were displayed here. RAV221 and RAV868 indicate association with breast cancer while RAV832 is associated with colon and rectal cancer. (COAD: Colon Adenocarcinoma, BRCA: Breast Invasive Carcinoma, LUAD: Lung Adenocarcinoma, READ: Rectum Adenocarcinoma, UCEC: Uterine Corpus Endometrial Carcinoma) **b** Validation of TCGA-BRCA. From panel (**a**), we showed RAV221 is associated with breast cancer and confirmed RAV221 is one of the top validated RAVs for TCGA-BRCA. Top 5 validated RAVs (*score*, bottom panel) and their average silhouette width (*avg.sw*, top panel) are shown. **c** A word cloud of MeSH terms associated with RAV221. We collected MeSH terms assigned to the publications belonging to RAV221 and weighted them based on their prevalence and the contribution to any given RAV. This word cloud shows that RAV221 is heavily composed of principal components from studies of breast neoplasms. **d** Three studies contributing to RAV221. **e** Top 10 enriched pathways in RAV221.

To demonstrate the ability to match datasets under new analysis to relevant published datasets, we applied RAVmodel to five TCGA datasets (Fig. 2a). Based on the correlation to principal components of these datasets, we identified RAVs specific to breast invasive carcinoma (RAV221 and RAV868) and to colon and rectal adenocarcinoma (RAV832). When RAVmodel was applied to the Breast Invasive Carcinoma (TCGA-BRCA) dataset, RAV221 was assigned with the highest validation score (Fig. 2b, Supplementary Table 1) and the associated MeSH terms were mostly breast-related terms, such as ‘breast’ and ‘breast neoplasms’ (Fig. 2c, drawWordcloud function). We extracted three breast-cancer studies contributing to RAV221 (Fig. 2d, findStudiesInCluster function). GSEA annotations on RAVs were queried and the top 10 enriched pathways were all breast-cancer associated (Fig. 2e, subsetEnrichedPathways function). We also checked RAV832 on its association with Colon Adenocarcinoma (TCGA-COAD) and Rectum Adenocarcinoma (TCGA-READ) datasets. RAV832 was assigned with the second-highest validation score for both COAD and READ datasets (Supplementary Fig. 6a, 7b, respectively) and contained MeSH terms such as ‘colorectal cancers’, ‘colon’, and ‘adenocarcinoma’ (Supplementary Fig. 6c). We also recognized that three out of five training data in RAV832 directly represented colon-associated illnesses (Supplementary Fig. 6d) and the top enriched gene set was an upregulated pathway in colorectal adenoma (Supplementary Fig. 6e). In summary, we confirmed that RAVmodel serves as a specific and robust index, coherently connecting expression profile, gene sets, related studies and their associated metadata (Fig. 1b), ultimately enhancing the interpretation of new datasets in the context of existing databases.

### RAVs to characterize colorectal cancer

**Fig. 3:**
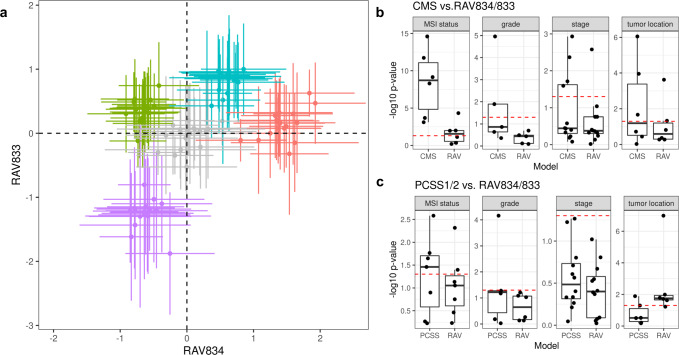
Sample scores for disease subtyping and metadata characterization. Sample scores from RAV834 and RAV833 were assigned to 3567 tumor samples from 18 colorectal carcinoma (CRC) studies. **a** All the sample from 18 datasets were assigned to either (i) one of the 4 previously proposed Consensus Molecular Subtypes (CMS) subtypes by CRC Subtyping Consortium (labeled with non-gray colors) or (ii) not assigned to a CMS subtype (gray), which comprised of 90 groups (5 subtype groups for 18 datasets). Each of these 90 groups is represented by the mean (point) and standard deviation (error bar) of sample scores. CMS subtypes separate when plotted against RAV834/833 coordinates. We further evaluated the capacity of RAVs to demonstrate clinicopathological characteristics of colon cancer. **b** Clinical phenotypes were regressed on discrete CMS subtypes and RAV834/833-assigned sample scores as covariates. Likelihood-ratio tests (LRTs) were used to compare the full model to a simplified model containing only CMS subtype (CMS, left box) or RAV834/833-assigned sample scores (RAV, right box) as predictors. RAV834/833-only model shows -log10p-value near 0, implying that CMS is not providing additional information. **c** The same regression and LRTs as in panel (**b**) were done using PCSS1/2 and RAV834/833-assigned sample scores as covariates. RAV834/833 outperforms PCSS1/2 on explaining colon cancer phenotypes except tumor location. Boxplot statistics are summarized in Supplementary Data 5 and raw data are included in Supplementary Data 6.

To compare the utility of GenomicSuperSignature relative to the focused use of data from a single disease, we compared RAVs to two previous studies that employed CRC gene expression databases to identify CRC molecular subtypes. The CRC Subtyping Consortium used 18 CRC datasets from multiple platforms comprising 4151 patients to define four discrete Consensus Molecular Subtypes (CMS) observed across numerous patient cohorts^19,20^. Ma et al. subsequently proposed a continuous scoring system called PC Cluster Subtype Scores (PCSS) based on an analysis of 8 CRC microarray datasets comprising 1,867 samples and found it was more closely correlated to microsatellite instability (MSI)^21,22^, grade, stage, and tumor location^19,20^. Importantly, these previous efforts both employed curated databases of only CRC transcriptomes, whereas the training set of the current RAVmodel consists of less than 2% CRC studies (Supplementary Data 2). We identified the RAVs most highly associated with CMS subtypes (RAV834/833) and PCSSs (RAV1575/834) (Supplementary Note 3) and confirmed that these RAV pairs showed comparable or higher performance on colon cancer subtyping than CRC subtyping efforts defined by bespoke methods in focused datasets (Fig. 3a, Supplementary Fig. 7a).

Using training and validation data of the original CRC studies, we compared associations between different subtype models and RAVs with the same clinicopathological variables. Notably, these data were not part of RAV training and are microarray datasets whereas the RAVs were trained exclusively from RNA-seq data. We used the likelihood-ratio test (LRT) to compare the different subtype models for association with clinicopathological variables^19^. A *p*-value near 1 (−log_10_*p*-value near 0) means that no additional information is provided by a full model composed of two subtype definitions compared to a model with only one. CMS-associated RAVs performed better than discrete CMS on all four phenotypes and also outperformed PCSSs except for tumor location (Fig. 3b, c). Interestingly, PCSS-associated RAVs were still better than CMS but slightly worse than PCSSs, while CMS-associated RAVs were better than both CMS and PCSSs, indicating that RAVs contain more comprehensive information than PCSSs (Supplementary Fig. 7b, c). This superior performance became more significant using only the 10 original validation datasets, excluding 8 datasets used to train the PCSS model (Supplementary Fig. 8). In conclusion, RAVs trained from heterogeneous datasets, not specific to CRC, captured biologically relevant signatures for CRC as well or superior to focused efforts using CRC-specific databases, suggesting that RAVs are of general use and can be applied to describe other diseases as well.

### Identifying common biological attributes across different datasets

**Fig. 4:**
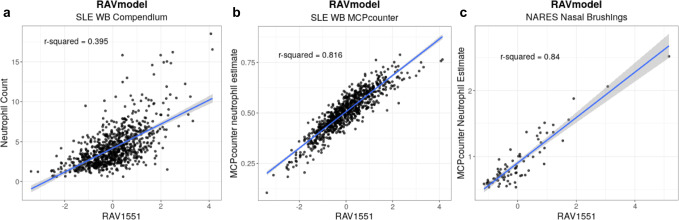
Estimate biological features of a new dataset using the signatures learned from public databases. RAVs encompass biological signals applicable across different platforms and independent datasets. We demonstrate this transfer learning capacity of RAVs by identifying the neutrophil-associated RAV from systemic lupus erythematosus whole blood (SLE-WB) data and using the same RAV to analyze nasal brushing (NARES) dataset. **a** Neutrophil counts of 853 samples from the SLE-WB dataset were plotted against RAV1551-assigned sample scores. **b** Neutrophil count estimates by MCPcounter were plotted against sample scores assigned by RAV1551. **c** Neutrophil count of 76 NARES samples were estimated by MCPcounter and plotted against RAV1551-assigned sample scores. The shaded area is the 95% confidence interval for predictions from a linear model.

For practical and technical reasons, biological datasets often contain missing information or signals buried in noise. GenomicSuperSignature can fill out those gaps by uncovering weak or indirectly measured biological attributes of a new dataset by leveraging the existing databases. To evaluate this transfer learning aspect of the GenomicSuperSignature, we compared the neutrophil count estimation by RAVs across two different datasets^8^ - systemic lupus erythematosus whole blood (SLE-WB)^23^ and nasal brushing (NARES)^24^ datasets. We searched for the SLE pathology-relevant RAV in three different ways using the SLE-WB dataset^23^. First, we identified RAV1551 based on the highest validation score with the positive average silhouette width. Second, we searched for the keyword, *neutrophil*, in the top three enriched pathways of all RAVs. Thirteen RAVs, including RAV1551, had two keyword-containing enriched pathways. Lastly, we used the neutrophil count of the SLE-WB dataset to find the metadata-associated RAV. For the continuous variables like neutrophil count, we compared the R^2^ between the target variable and all RAVs, where RAV1551 showed the maximum *R*^2^, 0.395 (Fig. 4a). A neutrophil is a terminally differentiated cell type and potentially under-detected in the active gene expression profile, so we used the neutrophil estimate from MCPcounter^25^ and further evaluated the correlation between the RAV1551 score and neutrophil estimate^8^. A stronger correlation between the RAV1551 score and the neutrophil estimate was observed (Fig. 4b). We concluded that RAV1551 is the SLE pathology-relevant RAV, specifically associated with the neutrophil counts, and tested whether this information can be expanded beyond the SLE dataset. For that, we applied RAV1551 on the NARES dataset, which is a gene expression profile of nasal brushings obtained from granulomatosis in polyangiitis (GPA) patients, a condition that causes inflammation of blood vessels affecting ears, noses, throats, lungs, and kidneys^24^. RAV1551 was not a top validated signal, ranked 14th with the validation score 0.41 with PC1 of NARES dataset, implying that neutrophil phenotype is not a major feature of this dataset. However, R^2^ between the neutrophil estimate of NARES dataset and RAV1551 score was 0.84 (Fig. 4c). This suggests that RAV can serve as a new measure to compare different datasets and provide an interpretation of potentially subtle biological signals (see Supplementary Notes 4 and 5 for additional examples).

## Discussion

A key innovation of GenomicSuperSignature is the creation of RAVindex consisting of principal components repeatedly observed in independent analysis of multiple published datasets (Fig. 1a). Compared to approaches that merge training data, this strategy is highly scalable, can identify latent variables specific to small training datasets, and ignores technical artifacts that are not observed across multiple datasets. The RAVindex is annotated with publication citations, MeSH terms, and gene sets, all of which are stored as the ‘RAVmodel’. Assembly of this information through the RAVmodel creates an information resource that can be rapidly applied to new datasets on a standard laptop (Fig. 1b). GenomicSuperSignature augments standard transcriptomic exploratory data analysis by providing modes of interpretation and hypothesis testing that were previously impractical to apply.

GenomicSuperSignature contains information learned from a large body of existing studies that can be “transferred” to newly collected data. For example, the RAVindex contains cancer type-specific RAVs (Fig. 2a), including RAVs that are more closely related to clinicopathological variables of CRC than the transcriptome subtypes previously identified through intensive analysis of CRC-specific databases bespoke subtyping efforts (Fig. 3, Supplementary Fig. 7). Such transfer learning is broadly applicable but particularly beneficial to the study of rare diseases and to small datasets where weak and under-represented, but biologically meaningful, signals can be identified^8^. To demonstrate this, we identified a RAV that was highly correlated to neutrophil content using SLE-WB dataset not in the model training data and used this RAV to estimate the neutrophil content in NARES dataset that lacks neutrophil count information (Fig. 4). In addition to data inference, GenomicSuperSignature can be useful for analyzing disease progress, comparing phenotypes across independent datasets, and identifying weak biological signals. The current RAVindex contains 4764 RAVs and 3386 out of them are observed in two or more independent datasets, which can be expected to have many other such applications through transfer learning.

GenomicSuperSignature is expected to be robust to batch effects because it characterizes clusters of highly similar latent variables from two or more independent studies, enabling it to ignore any signatures that are unique to a single study. We demonstrated the robustness of GenomicSuperSignature through sensitivity analysis and benchmarking against prior disease-specific analyses^8,19^. While trained exclusively on RNA-seq datasets, the performance of GenomicSuperSignature was not diminished when applied to microarray datasets. Furthermore, we observed transfer learning functionality compatible with the results from recount2-MultiPLIER^8^, even though RAVmodel was built via a different matrix decomposition method, was trained at the sample level instead of dataset level, and used training datasets with only ~10% overlap of samples (Supplementary Table 3). We conclude that GenomicSuperSignature is robust to different technological platforms and to the heterogeneity of training datasets, and enables interpretation of divergent datasets without subject-specific models.

GenomicSuperSignature offers significantly improved usability over the existing tools by adopting user-friendly application schemas. First, the pre-built models greatly reduce computational requirements for users: whereas training the current model took several days on 24 cores with 128 Gb memory, its application can be performed in seconds on a conventional laptop computer. Its implementations as an R/Bioconductor package^26^ and a Galaxy tool^27^ allow ready incorporation into widely used RNA-seq analysis pipelines and enables a large research community to reuse public data for more accurate analyses of new data.

The approach taken for GenomicSuperSignature is flexible and can be extended to other large publicly available databases. We plan to develop RAVmodels trained on microarray, single-cell RNA sequencing, and spatial transcriptomic data, and extend the approach to model organisms and metagenomic data from microbiome studies. Cross-species RAVmodels can help extend the discoveries from model organisms to humans^28^. These planned efforts will generate an expanded information resource with broader applicability and enhanced utility. For example, the GSEA annotation part of the model is independent of the RAVindex building process, so we can easily build multiple versions of RAVmodel with different gene sets or even any combination of gene sets. Also, we can expand RAVmodel with additional information on the training data because RAVs maintain the information on their source data. While the collection of RAVmodels grows as described, the GenomicSuperSignature package will be maintained as a stand-alone toolbox equally applicable to different RAVmodels. GenomicSuperSignature and its associated data resources will provide biomedical researchers with a new set of data exploration tools exploiting knowledge gained from hundreds and eventually thousands of existing public datasets.

## Methods

### Source data

We used human RNA sequencing datasets from RNA-seq Sample Compendia in refine.bio^17^, which hosts uniformly processed gene expression data from EBI’s ArrayExpress, NCBI’s GEO, and SRA. Data were downloaded on April 10th, 2020, and the datasets for model training were selected based on the following criteria: (1) Exclude studies with more than 1,000 samples because they are more likely to be single-cell RNA sequencing datasets. (2) Exclude studies assigned with a MeSH term, “Single-Cell Analysis”. (3) Exclude studies with fewer than 50 successfully downloaded and imported samples (Supplementary Data 1, Supplementary Fig. 2). Criteria 1 and 2 are not meant to entirely eliminate single-cell data but do serve to reduce the chance of including large, sparse datasets for which we plan to develop more specialized approaches. After filtering, the complete compendium includes 536 studies (defined as a single SRA study; Supplementary Data 2) comprising 44,890 samples.

#### Processing training datasets

Training data included each sample’s quant.sf file from Salmon outputs^29^, not aggregated or normalized. We imported quant.sf files using tximport, scaling transcripts-per-million (TPM) using the average transcript length across samples and the library size (“lengthScaledTPM”)^30^, followed by the log2 transformation. Ensembl transcript names were converted into gene symbols using the AnnotationDbi package^31^. Row normalization was done on all samples together, not at the individual study level, because correcting variability at the study level could remove the signals we want to capture^32^. For model building, we used 13,934 common genes among 536 studies’ top 90% varying genes, where the variation cutoff was based on their study-level standard deviation (Supplementary Fig. 1a).

#### Build RAVmodels

We performed PCA on pre-processed gene expression matrices independently for each study using the stats::prcomp R function for each gene, centered but not scaled. Loading vectors of the top 20 PCs from 536 studies (total 10,720 PCs) were clustered via hierarchical clustering. For hierarchical clustering, we calculated the distances between loadings using Spearman’s correlation coefficient and clustered them with the ward.D agglomeration method. The number of clusters was set to the minimum that can separate up to 50 negative controls (Supplementary Fig. 3). PCs in each cluster were averaged and the resulting ‘genes x averaged loadings’ matrix, RAVindex, was combined with associated metadata, GSEA, and MeSH annotations into a unified data structure that we term a PCAGenomicSignatures (Supplementary Fig. 1b). The following sections detail extensive sensitivity analyses and optimizations to choose appropriate modeling approaches and parameters.

##### Datasets for method choice and optimization

During the optimization process for RAVmodel building, we used the small, well-characterized datasets: 8 colon cancer datasets from curatedCRCData^33^, 10 ovarian cancer datasets from curatedOvarianData^34^, and recount2 datasets^35^ used for the recount2-MultiPLIER model^8^. Training datasets for the recount2-MultiPLIER model and the current version of RAVmodel are partially overlapping: the recount2-MultiPLIER model used 37,027 runs from 30,301 unique samples from 1466 studies and GenomicSuperSignature was constructed from 44,890 runs from 34,616 unique samples from 536 studies. Among them, only 6839 runs from 5260 unique samples from 87 studies were used by both models. In addition to the different combinations of these datasets, we created synthetic datasets that served as positive and negative controls.

##### Dimensionality reduction methods

We assessed multiple dimensionality reduction methods for RAVindex building. Non-negative Matrix Factorization (NMF) was excluded because there is no clear criterion to select representative components, such as variance explained by each principal component in PCA. Non-orthogonal relationship between components captured by NMF is potentially a more relevant representation of biological data, but by combining replicative principal components, we overcome the orthogonality constraint imposed by PCA. We also ruled out independent component analysis (ICA) because it separates independent signals to reduce the effect of noise or artifacts^36^, which is different from our goal to extract biological signals, and, like NMF, it also does not rank its components. We, therefore, selected two dimensionality reduction methods, PCA and PLIER^7^, and investigated the types of signatures when they were applied at the dataset level or sample level. This comparison was done across four different conditions: perPCA (PCA on each dataset and cluster top PCs), megaPCA (PCA on all samples), perPLIER (PLIER on each dataset and cluster latent variables (LVs, equivalent to principal components from PCA)), and megaPLIER (PLIER on all samples, identical to MultiPLIER). One of the downsides of the megaPLIER approach was that the direct link between LVs and the training data was not available. Also, the annotation database was inseparable from the model building, making it harder to scale. The perPLIER approach blended LVs in each cluster and lost distinct signatures. Like megaPLIER, megaPCA did not maintain the links between signature and its source data. Additionally, megaPCA picked up only a handful of strong signatures in top PCs, which we can still capture through the perPCA approach without losing weaker signatures. Overall, we choose the perPCA approach for our model building because it is more scalable, keeps the link between signature and its source data, and captures both pan-dataset and per-dataset signatures.

##### Data transformation

We applied log2 transformation and row normalization across all samples, not at the dataset level, to maintain the differences in scale between the datasets. Variance-Stabilizing Transformation (VST) was excluded because it requires significantly more computing resources - over 200 times longer user CPU times, without any meaningful improvement on capturing biological signatures over log2 transformation because we removed low variable genes from our training datasets^37^.

##### Subset genes

We searched for the minimum set of genes carrying the replicable biological signals, because more genes require more computing resources to process and some genes were measured only in certain training datasets. Also, low- or non-expressing genes can be indistinguishable from the background noise and including them could make interpretation harder. First, we examined the models built from two sets of common genes subset at the different entities—among training datasets versus between training datasets and annotation databases. The model using the common genes from both training datasets and annotation databases didn’t improve the accuracy of GSEA compared to the the other and made the model building process less scalable because RAVindexes need to be rebuilt for RAVmodels with different annotation databases even for the same training datasets. Next, instead of using a fixed cutoff for ‘low-expressing’ genes, we selected genes based on their expression variance within the dataset because we suspect that genes with a stable expression level within a dataset convey less information to capture. So for the RAVmodel building, we used the common genes among the top 90% varying genes from each training dataset.

##### The number of PCs to collect

We decided on the number of PCs to collect based on the following four reasons. First, a threshold of 20 PCs is adequate for stability of the RAV model, particularly for larger clusters of 3+ PCs. Most of the lower PCs (PC11-20) are in single-element (22%) or two-element (53%) clusters. We expect further relaxing the cutoff would contribute even less to clusters of 2+ size, which we validate using two RAVmodels consisting of (1) top 10 PCs (RAVmodel_10) or (2) top 20 PCs (RAVmodel_20) from each dataset. We chose the most similar pairs of RAVs between 2382 RAVs (RAVmodel_10) and 4764 RAVs (RAVmodel_20) using the Pearson coefficient. 79% of RAVs in RAVmodel_10 have a similar or identical RAVs in RAVmodel_20 with the Pearson coefficient > 0.7 and the average Pearson coefficient for the RAVs with more than 2 elements is 0.86, suggesting that the model is robust to cutoffs of > = 10 PCs, and that the added computational cost of a cutoff larger than 20 would provide little or no benefit. Second, we chose the top 20 PCs because they represent a majority of the gene expression variance in each study—the median percentage of total variability represented 63%. Third, we applied the elbow method to find the number of ‘significant’ PCs to collect, using the num.pc function implemented in the PLIER package with the following modifications^7^. The PLIER::num.pc function applies z-score normalization, but because our method does normalization with all samples combined, we removed this internal normalization from PLIER::num.pc and provided the pre-normalized input data instead. The number of significant PCs from this modified PLIER::num.pc function ranged from 5 to 45, while the “elbow” of the scree plots was not always clear on manual inspection. We chose the median value, 20, as a pre-set cutoff for the different training datasets. Using a varying number of PCs would add complexity to the process that seemed unjustified given that the variance explained by each PC does not vary much by study size, ranging from 50 to 100 for our 536 training datasets. For example, after the 8th PC, less than 5% of the variance was explained by a single PC for all 536 training datasets—the maximum variance explained by PC7 and PC8 are 5.1% and 4.6%, respectively. Finally, one of the main works we benchmarked against was Ma et al., where the authors selected the top 20 PCs for their model building to extract colon cancer-specific signatures.

##### Synthetic datasets as a negative control

We used the negative-control dataset to explore different clustering methods and the optimum number of clusters for hierarchical clustering. First, we constructed 50 synthetic datasets by randomly selecting 50 samples from 44,890 samples. We scrambled genes in each of 50 synthetic datasets and added random values between −0.1 and 0.1. The mean and standard deviation of 44,890 samples were used for row normalization of the synthetic datasets. We confirmed that these synthetics datasets can serve as a negative control based on the distance matrix: the minimum and maximum distance of PC1s from the synthetic datasets ranged approximately between 1st and 3rd quarters of the distance distribution of PCs from the actual training datasets, which we want to separate during the clustering process (Supplementary Fig. 3a).

##### Clustering methods

To group the replicative PCs, we tried centroid-based clustering such as k-means and graph-based clustering, and connectivity-based clustering like hierarchical clustering. We applied them on the top 20 PCs from 8 colon cancer datasets and for evaluation, compared the cluster membership with the previously identified signatures (PCSSs) using the Jaccard index. We also applied the different clustering methods on the top 5 PCs from 10 positive and 10 negative controls, which were synthetic datasets created through bootstrap and random sample selection, respectively. We evaluated each clustering method based on how often the top PCs from positive controls were clustered together and the top PCs from negative controls were assigned to different clusters.

For the centroid-based clustering methods (k-means and k-medoids), we searched the optimum number of clusters using multiple measures including the elbow method, mean silhouette width, and within-cluster sum of squares. However, the number of clusters required to separate unrelated PCs was too high to keep the related PCs together, which could not be improved with different distance metrics. We suspect that PCs from biological data do not possess a spherical or ellipsoidal symmetry required for centroid-based clustering to work.

We evaluated graph-based clustering and hierarchical clustering with the different combinations of distance metrics and agglomeration methods (for hierarchical clustering) on the same datasets used for the centroid-based clustering tests. From this evaluation, the clustering schema was narrowed down into two versions: graph-based edge-betweenness clustering using edge weight by Spearman correlation, and hierarchical clustering using Spearman distance and ward.D agglomeration. When we applied graph-based clustering approaches to larger datasets, however, there were scalability issues: it formed a very large cluster, containing more than 5% of all PCs, that failed to group even the positive control PCs due to the extreme distribution of the cluster sizes. So we decided to use hierarchical clustering based on Spearman distance with ward.D agglomeration.

##### Choose the optimum number of clusters for hierarchical clustering

We collected the top 20 PCs from 536 training datasets and PC1s from varying numbers of negative-control, synthetic datasets (10, 20, 30, 40, and 50) and performed hierarchical clustering with the different numbers of clusters. Nine different cluster numbers were applied to each datasets: those nine cluster numbers were round({#ofPCs}/*d*), where *d* is 7, 6, 5, 4, 3, 2.75, 2.5, 2.25, and 2. All negative controls were separated when *d* = 2.25, regardless of the number of negative controls (Supplementary Fig. 3b, c). So for the current versions of RAVmodel, we selected 4764 clusters (= round((20 × 536)/2.25)).

##### Model validity

To test whether heterogeneous datasets can maintain the signatures from the focused dataset, we first built RAVindex from the focused training datasets and gradually “contaminated” the training datasets with the unrelated datasets. A rate of overlapping enriched pathways over correlated pathways was monitored, from which we confirmed that our RAVindex building process reliably maintains the dataset-specific signatures from the heterogeneous training datasets.

#### Annotate RAVs with gene sets

Gene Set Enrichment Analysis (GSEA) is a common approach used to supply biological interpretation to lists or sets of genes^38–40^ that has also been used to interpret biological signals in principal components^41^. We subjected each RAV to GSEA to aid in interpreting the biological signals associated with it. Genes were ordered by loading value from each RAV and supplied as a geneList input for clusterProfiler::GSEA^42^. We filtered enriched pathways with Benjamini–Hochberg (BH) adjusted *p*-value < 0.05 and among them, collected the pathways with the minimum *q*-values. The subset of GSEA results—NES, Description, pvalue, and qvalues - were included in the RAVmodel. RAVmodels used in this study are (1) RAVmodel_C2, which was annotated with Molecular Signatures Database (MSigDB) curated gene sets (C2, version 7.1)^38,43^, excluding any MSigDB C2 gene set with fewer than 10 genes or more than 500 genes, and (2) RAVmodel_PLIERpriors annotated with the three prior gene sets (bloodCellMarkersIRISDMAP, cannonicalPathways, and svmMarkers) provided through the PLIER package^7^ (Supplementary Table 2).

#### Annotate RAVs with MeSH terms

MeSH terms^44^ were assigned to each study using the NCBI Medical Text Indexer (MTI) tool^45^. The relevance of MeSH terms in each RAV was assessed through the bag-of-words model: all the MeSH terms associated with the training datasets were considered as the ‘universe’ and each term in the cluster was reverse-weighted by the frequency of the given term in the universe. MeSH terms were also weighted by the variance explained by the principal component that they came from. The significance of MeSH terms associated with each cluster was evaluated based on their exclusivity. However, the simple sum of associated MeSH terms can be inappropriate in some cases. For example, noise can be a predominant signal in small clusters and common MeSH terms, such as ‘human’ or ‘RNA sequencing’ for the current version of RAVmodel, can be overrepresented and silence the other terms. To handle these extreme situations, we incorporated additional filtering and normalization terms. If the cluster contains less than 8 PCs, we considered any MeSH terms appearing half down of ‘cluster size × 0.5’ as noise and removed them. If the cluster has more or equal to 8 PCs in it, any MeSH terms appearing less than or equal to 4 times were eliminated. These cutoff values for ‘noise’ can be customized by users to fit their needs. We also provide the option to exclude potentially non-informative terms due to the lack of specificity (e.g. ‘Human’ and ‘RNA sequencing’ for the current model) as the ‘dropList’ in the GenomicSuperSignature package, which is customizable as well. The remaining MeSH terms were scored as the sum of the variance explained by PCs divided by the frequency of that term in the universe. This final score can be displayed as a table or a word cloud, using meshTable or drawWordcloud functions, respectively.

#### Input datasets for validation

The GenomicSuperSignature can be applied to gene expression profiles generated from both microarray and RNA sequencing with the minimum pre-processing. The major requirement for inputs is that the gene expression profile should approximate a normal distribution.

For validation, we used five TCGA RNA sequencing datasets (COAD, BRCA, LUAD, READ, and UCEC) acquired from GSEABenchmarkeR^40^. Any genes with count-per-million (CPM) less than 2 were excluded, and the count matrix was log2-transformed and centered but not scaled before PCA. Eighteen colon cancer microarray datasets from curatedCRCData were also used for validation^46^. Missing and infinite values were removed from these microarray data and the remaining expression values were centered at each gene level. To evaluate the transfer learning capacity, we used the pre-processed versions of NARES^24^ and SLE-WB datasets^23^.

### Reporting summary

Further information on research design is available in the Nature Research Reporting Summary linked to this article.

## Supplementary information

Supplementary Information

Editorial Assessment Report

Description of Supplementary Data

Supplementary Dataset 1

Supplementary Dataset 2

Supplementary Dataset 3

Supplementary Dataset 4

Supplementary Dataset 5

Supplementary Dataset 6

Supplementary Dataset 7

Supplementary Dataset 8

Reporting Summary

## Data Availability

The data analyzed in this study are available in Zenodo with the identifier [10.5281/zenodo.6496611]^47^. Training datasets used for the current RAVmodel are available at refine.bio RNA-seq sample compendia (https://www.refine.bio/compendia?c=rna-seq-sample). Study accession numbers for the model training datasets are listed under the ‘studyName’ column of Supplementary Data 1. The source data for a given training dataset can be accessed and freely downloaded from the following link: https://www.refine.bio/experiments/{studyNames}.

## Code availability

The workflow to build the RAVmodel is available from https://github.com/shbrief/model_building which is archived in Zenodo with the identifier [10.5281/zenodo.6496552]^48^. All analyses presented here are reproducible using code accessible from https://github.com/shbrief/GenomicSuperSignaturePaper/ and archived in Zenodo with the identifier [10.5281/zenodo.6496612]^47^. GenomicSuperSignature package for this work is available at [10.18129/B9.bioc.GenomicSuperSignature]^49^.

## References

[1] Meng C (2016). Dimension reduction techniques for the integrative analysis of multi-omics data. Brief. Bioinform..

[2] Myers CL, Barrett DR, Hibbs MA, Huttenhower C, Troyanskaya OG (2006). Finding function: evaluation methods for functional genomic data. BMC Genomics.

[3] Newman AM (2015). Robust enumeration of cell subsets from tissue expression profiles. Nat. Methods.

[4] Leek JT (2010). Tackling the widespread and critical impact of batch effects in high-throughput data. Nat. Rev. Genet..

[5] Stein-O’Brien GL (2019). Decomposing cell identity for transfer learning across cellular measurements, platforms, tissues, and species. Cell Syst..

[6] Sharma, G., Colantuoni, C., Goff, L. A., Fertig, E. J. & Stein-O’Brien, G. projectR: An R/Bioconductor package for transfer learning via PCA, NMF, correlation, and clustering. *Bioinformatics*10.1093/bioinformatics/btaa183 (2020). 10.1093/bioinformatics/btaa183 PMC7267840 32167521

[7] Mao W, Zaslavsky E, Hartmann BM, Sealfon SC, Chikina M (2019). Pathway-level information extractor (PLIER) for gene expression data. Nat. Methods.

[8] Taroni JN (2019). MultiPLIER: a transfer learning framework for transcriptomics reveals systemic features of rare disease. Cell Syst..

[9] Korsunsky I (2019). Fast, sensitive and accurate integration of single-cell data with Harmony. Nat. Methods.

[10] Sastry AV (2021). Independent component analysis recovers consistent regulatory signals from disparate datasets. PLoS Comput. Biol..

[11] Lee J, Oh S, Sael L (2018). GIFT: guided and interpretable factorization for tensors with an application to large-scale multi-platform cancer analysis. Bioinformatics.

[12] Srivastava D, Iyer A, Kumar V, Sengupta D (2018). CellAtlasSearch: a scalable search engine for single cells. Nucleic Acids Res..

[13] Hao Y (2021). Integrated analysis of multimodal single-cell data. Cell.

[14] Peng, M., Li, Y., Wamsley, B., Wei, Y. & Roeder, K. Integration and transfer learning of single-cell transcriptomes via cFIT. *Proc. Natl Acad. Sci. USA* **118**, e2024383118 (2021). 10.1073/pnas.2024383118 PMC7958425 33658382

[15] Pan SJ, Yang Q (2010). A survey on transfer learning. IEEE Trans. Knowl. Data Eng..

[16] Liberzon A (2011). Molecular signatures database (MSigDB) 3.0. Bioinformatics.

[17] refine.bio. *Refine.bio* https://www.refine.bio.

[18] Love MI, Huber W, Anders S (2014). Moderated estimation of fold change and dispersion for RNA-seq data with DESeq2. Genome Biol..

[19] Ma S (2018). Continuity of transcriptomes among colorectal cancer subtypes based on meta-analysis. Genome Biol..

[20] Guinney J (2015). The consensus molecular subtypes of colorectal cancer. Nat. Med..

[21] Nojadeh JN, Behrouz Sharif S, Sakhinia E (2018). Microsatellite instability in colorectal cancer. EXCLI J..

[22] Ogino S, Goel A (2008). Molecular classification and correlates in colorectal cancer. J. Mol. Diagn..

[23] Banchereau, R., Hong, S., Cantarel, B. & Baldwin, N. Personalized immunomonitoring uncovers molecular networks that stratify lupus patients. *Cell* **165**, 551–565 (2016). 10.1016/j.cell.2016.03.008 PMC5426482 27040498

[24] Grayson PC (2015). Brief report: defining the nasal transcriptome in granulomatosis with polyangiitis (Wegener’s). Arthritis Rheumatol..

[25] Becht E (2016). Estimating the population abundance of tissue-infiltrating immune and stromal cell populations using gene expression. Genome Biol..

[26] Huber W (2015). Orchestrating high-throughput genomic analysis with bioconductor. Nat. Methods.

[27] Afgan E (2018). The Galaxy platform for accessible, reproducible and collaborative biomedical analyses: 2018 update. Nucleic Acids Res..

[28] Brubaker, D. K. et al. An interspecies translation model implicates integrin signaling in infliximab-resistant inflammatory bowel disease. *Sci. Signal*. **13**, eaay3258 (2020). 10.1126/scisignal.aay3258 PMC7459361 32753478

[29] Patro R, Duggal G, Love MI, Irizarry RA, Kingsford C (2017). Salmon provides fast and bias-aware quantification of transcript expression. Nat. Methods.

[30] Soneson C, Love MI, Robinson MD (2015). Differential analyses for RNA-seq: transcript-level estimates improve gene-level inferences. F1000Res..

[31] Pages, H., Carlson, M., Falcon, S. & Li, N. AnnotationDbi: annotation database interface. *R package version 1. 4* (2008).

[32] Lee, A. J., Park, Y., Doing, G., Hogan, D. A. & Greene, C. S. Correcting for experiment-specific variability in expression compendia can remove underlying signals. *Gigascience* **9**, giaa117 (2020). 10.1093/gigascience/giaa117 PMC7607552 33140829

[33] Parsana, P., Riester, M., Huttenhower, C. & Waldron, L. *curatedCRCData*. (Bioconductor, 2017). 10.18129/B9.BIOC.CURATEDCRCDATA.

[34] Ganzfried BF (2013). curatedOvarianData: clinically annotated data for the ovarian cancer transcriptome. Database.

[35] Collado-Torres L (2017). Reproducible RNA-seq analysis using recount2. Nat. Biotechnol..

[36] Yao F, Coquery J, Lê Cao K-A (2012). Independent principal component analysis for biologically meaningful dimension reduction of large biological data sets. BMC Bioinforma..

[37] Love MI, Anders S, Kim V, Huber W (2015). RNA-Seq workflow: gene-level exploratory analysis and differential expression. F1000Res..

[38] Subramanian A (2005). Gene set enrichment analysis: a knowledge-based approach for interpreting genome-wide expression profiles. Proc. Natl Acad. Sci. USA.

[39] Tilford CA, Siemers NO (2009). Gene set enrichment analysis. Methods Mol. Biol..

[40] Geistlinger, L. et al. Toward a gold standard for benchmarking gene set enrichment analysis. *Brief. Bioinform*. 10.1093/bib/bbz158 (2020). 10.1093/bib/bbz158 PMC7820859 32026945

[41] Frost HR, Li Z, Moore JH (2015). Principal component gene set enrichment (PCGSE). BioData Min..

[42] Yu G, Wang L-G, Han Y, He Q-Y (2012). clusterProfiler: an R package for comparing biological themes among gene clusters. OMICS.

[43] Liberzon A (2015). The Molecular Signatures Database (MSigDB) hallmark gene set collection. Cell Syst..

[44] Baumann N (2016). How to use the medical subject headings (MeSH). Int. J. Clin. Pract..

[45] Mork J, Aronson A, Demner-Fushman D (2017). 12 years on—is the NLM medical text indexer still useful and relevant?. J. Biomed. Semant..

[46] Parsana, P., Riester, M. & Waldron L. *curatedCRCData: Clinically Annotated Data for the Colorectal Cancer Transcriptome* (Bioconductor, 2022).

[47] Oh, S. *shbrief/GenomicSuperSignaturePaper: Release for Zenodo* (Zenodo, 2022). 10.5281/ZENODO.6496611.

[48] Oh S (2022). shbrief/model_building: Release Zenodo..

[49] Sehyun Oh <shbrief@gmail.com> [aut, cre], Levi Waldron [aut], Sean Davis <seandavi@gmail. com> [aut]. GenomicSuperSignature. (Bioconductor, 2021). 10.18129/B9.BIOC.GENOMICSUPERSIGNATURE.

