# Supplementary material for: GenomicSuperSignature facilitates interpretation of RNA-seq experiments through robust, efficient comparison to public databases
Source: Nat Commun. 2022 Jun 27;13:3695. doi: 10.1038/s41467-022-31411-3 (PMC9237024; doi:10.1038/s41467-022-31411-3)
Supplement: Supplementary file 1 — Supplementary Information [file 41467_2022_31411_MOESM1_ESM.pdf]

GenomicSuperSignature facilitates interpretation of RNA-seq experiments through robust, efficient comparison to public databases

Supplementary Figures **1**

- Supplementary Figure 1. Overview of RAVmodel building 1
- Supplementary Figure 2. Data availability from refine.bio 5
- Supplementary Figure 3. The optimum number of clusters 6
- Supplementary Figure 4. Distribution of RAV sizes 7
- Supplementary Figure 5. RAVs without enriched pathways 8
- Supplementary Figure 6. Colon and rectal cancer associated RAV 9
- Supplementary Figure 7. CRC characterization with different RAVs 10
- Supplementary Figure 8. CRC characterization with 10 validation datasets 11
- Supplementary Figure 9. Overview validation results via an interactive plot 12
- Supplementary Figure 10. PCA with GSEA annotation 13

Supplementary Tables **14**

- Supplementary Table 1. Summary of new terms 14
- Supplementary Table 2. Available RAVmodels 15
- Supplementary Table 3. Comparison between GenomicSuperSignature and MultiPLIER 16
- Supplementary Table 4. Comparison between GenomicSuperSignature and Seurat's weighted nearest neighbor 17

Supplementary Notes **18**

- Supplementary Note 1. Comparison to existing tools 18
- Supplementary Note 2. Software implementation 19
- Supplementary Note 3. RAVs for colorectal cancer characterization 20
- Supplementary Note 4. PCA plot annotated with pre-calculated GSEA 20
- Supplementary Note 5. Example of RAV interpretation 21

Supplementary References **22**

Supplementary Figures

Supplementary Figure 1. Overview of RAVmodel building

a We downloaded human `quant.sf` files from refinebio RNA-seq Sample Compendia. We subset studies with more than 50 and less than 1,000 samples from 6,460 studies available at the time of the snapshot. Some RAVmodels retain additional filtering criteria on their training datasets. For example, the current version of RAVmodel predominantly used in this study further excludes datasets potentially from single cell analysis. Selected datasets were imported through `tximport`, followed by log2 transformation to bring them close to normal distribution. We used the common genes among the top 90% varying genes of each study, which was 13,934 genes for the current RAVmodel, and did row normalization using mean and standard deviation calculated from all 44,890 samples. PCA was done on a row-normalized expression matrix at the study level and top 20 PCs from each study were collected, ending up with 10,720 PCs. Distance matrix between these PCs was calculated using Spearman correlation and hierarchical clustering was applied with the pre-defined optimum number of clusters. Weighted MeSH terms and GSEA on each RAV, along with RAVindex and other metadata, were assembled into PCAGenomicSuperSignatures object, named as RAVmodel. In the below workflow diagram, boxes represent the intermediate files we created during the model building process. **b** Schematic of PCAGenomicSignatures object. RAVindex is a 'genes x RAVs' matrix. The colData provides information on RAVs, such as studies contributing to each RAV and GSEA results from each RAV. The metadata stores details on the RAVmodel itself, such as cluster memberships of PCs and the size of each cluster. The trainingData provides information on studies used for the model training, which includes MeSH terms assigned to each study and PCA summary of each study. **c** User's perspective. The GenomicSuperSignature package allows users to access a RAVmodel (Z matrix, blue) and annotation information on each RAV. From a gene expression matrix (Y matrix, grey), users can calculate dataset-level validation score or sample score matrix (B matrix, red). Through the RAV of your interest, additional information such as related studies, GSEA, and MeSH terms can be easily extracted.

a

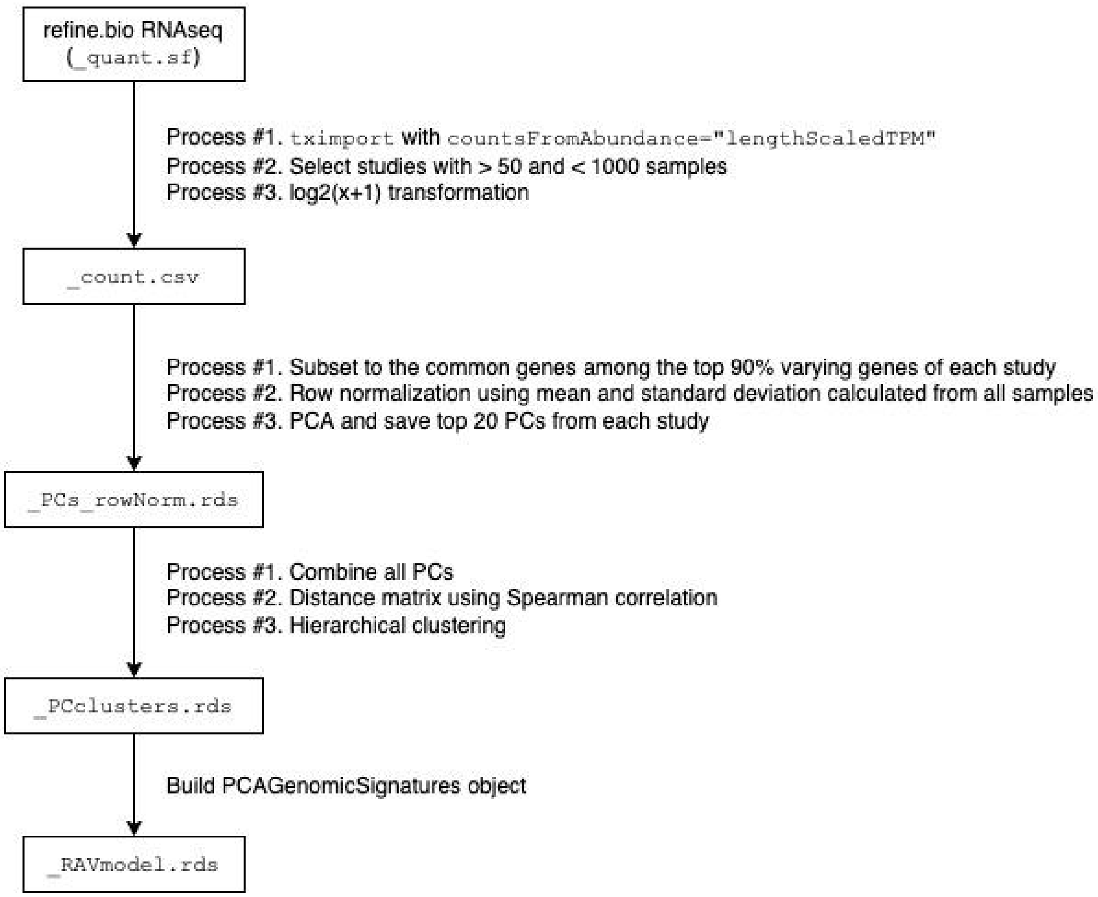
refine.bio RNAseq
(_quant.sf)
Process #1. tximport with countsFromAbundance="lengthScaledTPM"
Process #2. Select studies with > 50 and < 1000 samples
Process #3. log2(x+1) transformation
_count.csv
Process #1. Subset to the common genes among the top 90% varying genes of each study
Process #2. Row normalization using mean and standard deviation calculated from all samples
Process #3. PCA and save top 20 PCs from each study
_PCs_rowNorm.rds
Process #1. Combine all PCs
Process #2. Distance matrix using Spearman correlation
Process #3. Hierarchical clustering
_PCclusters.rds
Build PCAGenomicSignatures object
_RAVmodel.rds

b

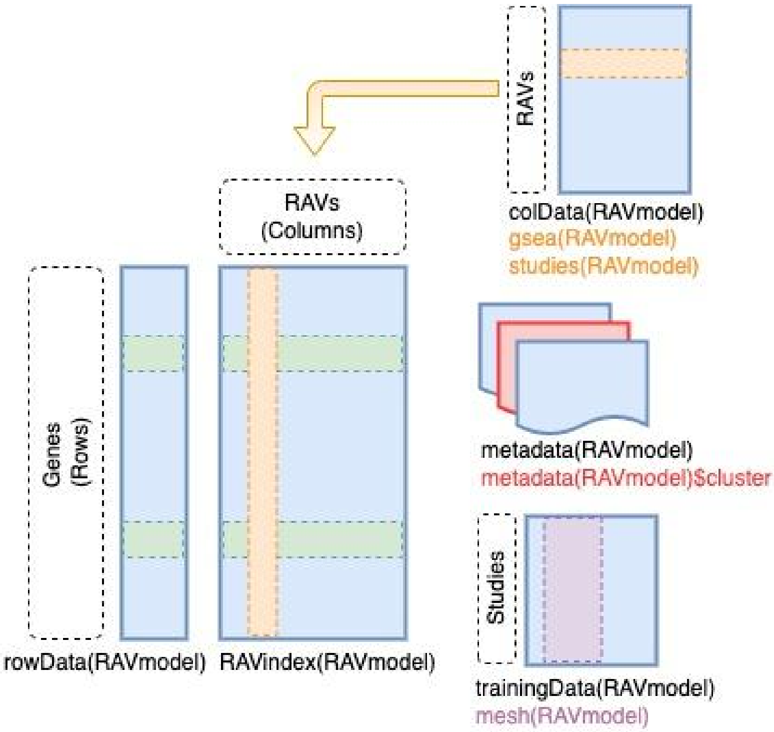
RAVs
RAVs
(Columns)
Genes
(Rows)
rowData(RAVmodel)
RAVindex(RAVmodel)
colData(RAVmodel)
gsea(RAVmodel)
studies(RAVmodel)
metadata(RAVmodel)
metadata(RAVmodel)$cluster
Studies
trainingData(RAVmodel)
mesh(RAVmodel)

c

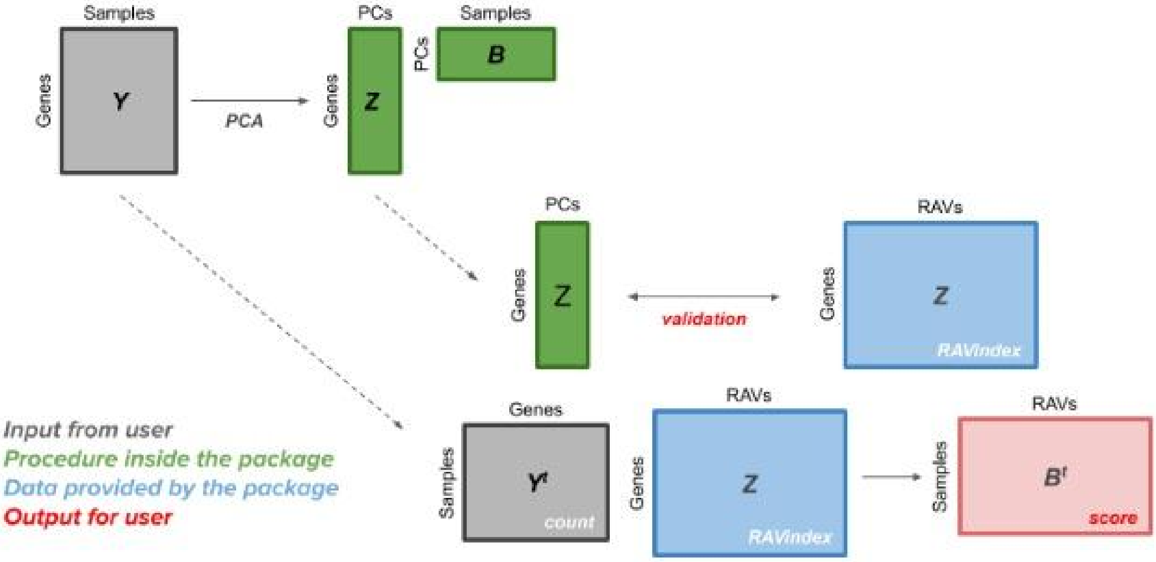
Samples
Genes
Y
PCA
PCs
Genes
Z
Samples
PCs
B
PCs
Genes
Z
validation
RAVs
Genes
Z
RAVindex
Input from user
Procedure inside the package
Data provided by the package
Output for user
Genes
Samples
Y^t
count
RAVs
Genes
Z
RAVindex
RAVs
Samples
B^t
score

Supplementary Figure 2. Data availability from refine.bio

The refinebio database is actively updated and our current RAVmodel is based on the snapshot on April 10th, 2020. Metadata bar (light blue) shows the number of studies with the given ranges of study sizes based on the metadata. Downloaded bar (pink) represents the number of studies with the given ranges of study sizes that were successfully downloaded and imported through `tximport`. Based on metadata, there were studies with more than 100 samples, but at the time of snapshot, only up to 100 samples were available. Thus, the plot displays only up to 100 samples/study cases. Due to the unavailability of certain samples, more studies belong to 0-5 samples/study bracket than metadata suggests.

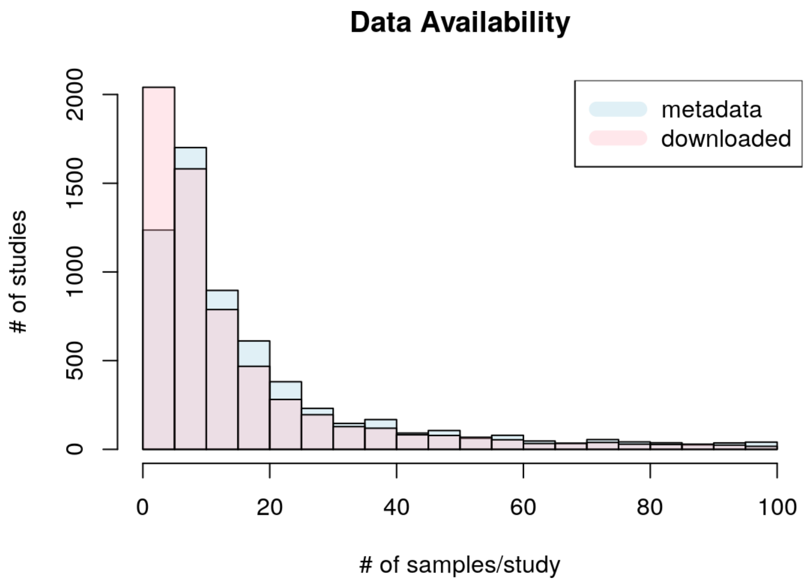

Supplementary Figure 3. The optimum number of clusters

We used PC1s from synthetic datasets, designed as negative controls, to decide the number of clusters for hierarchical clustering. Training datasets, top 20 PCs from 536 studies (`RAVmodel_536` column in Supplementary Data 1), were combined with PC1s from the different numbers of synthetic datasets (10, 20, 30, 40, and 50) (Supplementary Methods). **a** Heatmap of the distance matrix between 50 negative controls. Distance was calculated based on Spearman's correlation. **b** Proportion of the negative controls that were separated with the given cluster number. `numOfControls` is the number of negative controls added to the training datasets. `numOfCluster` is the round of the total PCs (from training datasets and negative controls) divided by 7, 6, 5, 4, 3, 2.75, 2.5, 2.25, and 2. Different numbers of negative controls were completely separated when we used the cluster number `k = round((the number of PCs)/2.25)`. **c** Number of negative controls that were not separated.

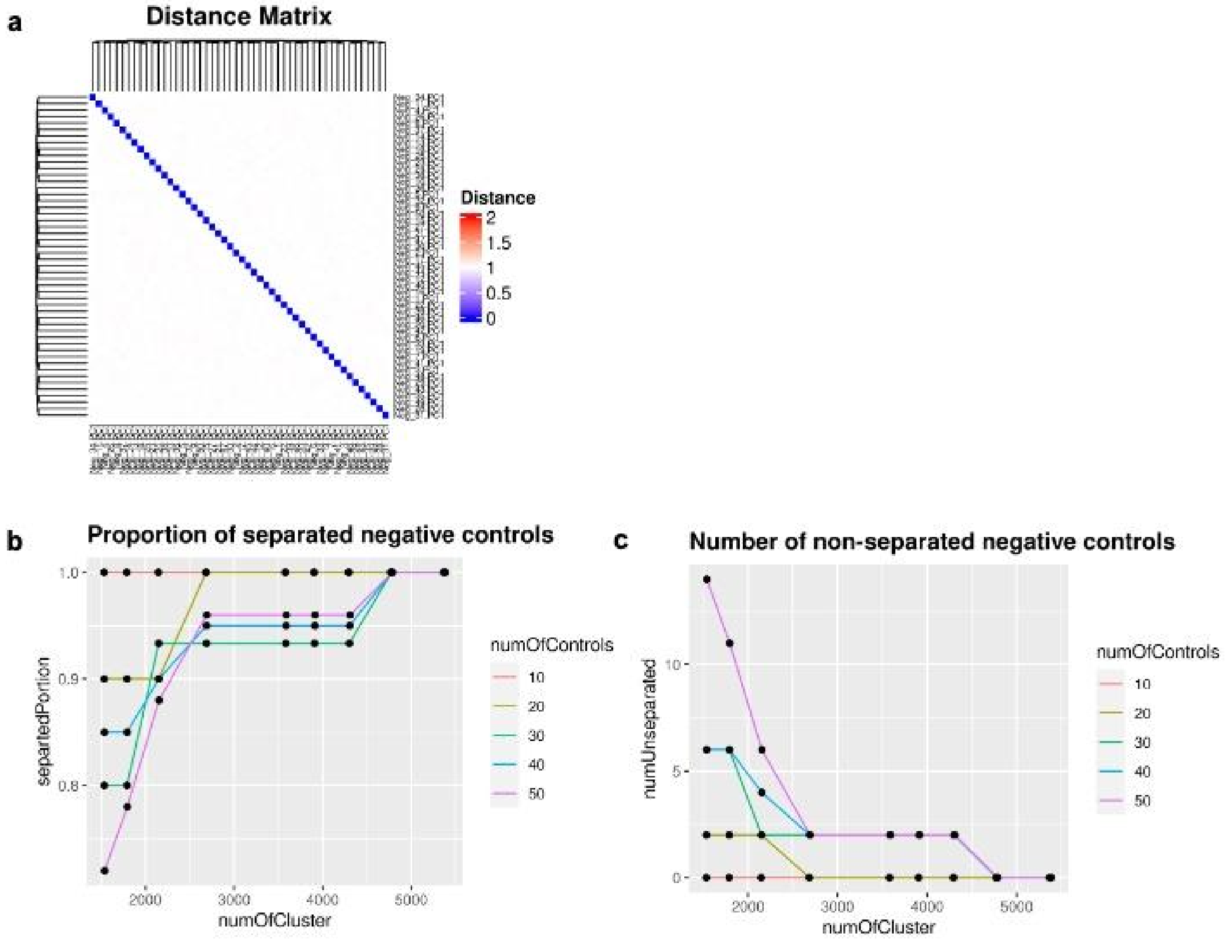

Supplementary Figure 4. Distribution of RAV sizes

RAVs are constructed from different numbers of PCs, ranging from 1 to 24. Here, we plotted the number RAVs (y-axis) against the cluster sizes (x-axis) to show the distribution of RAV sizes.

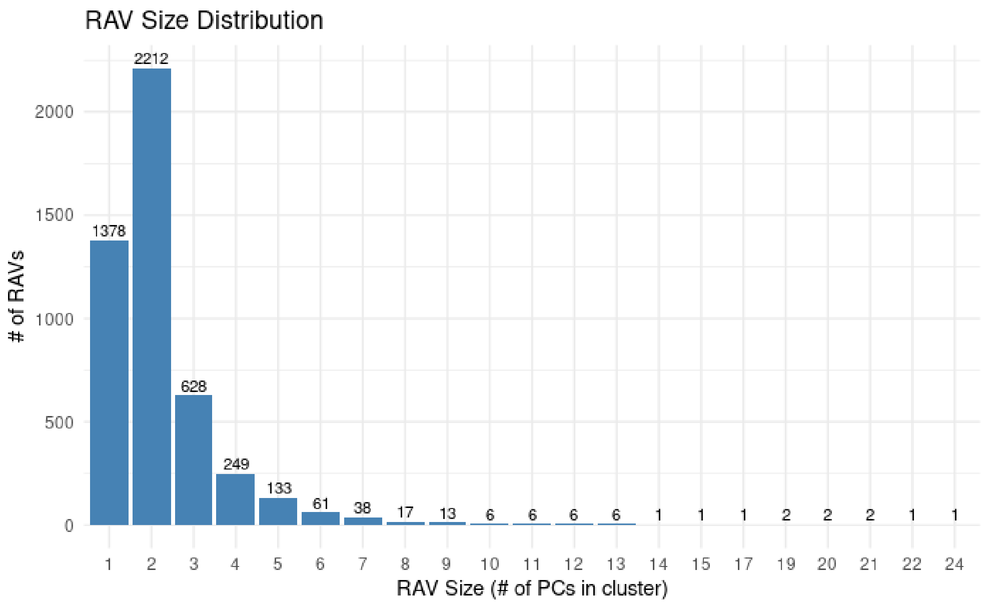

Supplementary Figure 5. RAVs without enriched pathways

We summarized the gene set annotation status of RAVs based on the RAV sizes. We tested two RAVmodels **(a)** RAVmodel annotated with MSigDB C2 and **(b)** RAVmodel annotated with three gene sets provided through the PLIER package. RAVs without enriched pathways are labeled with teal and RAVs with one or more enriched pathways are in red.

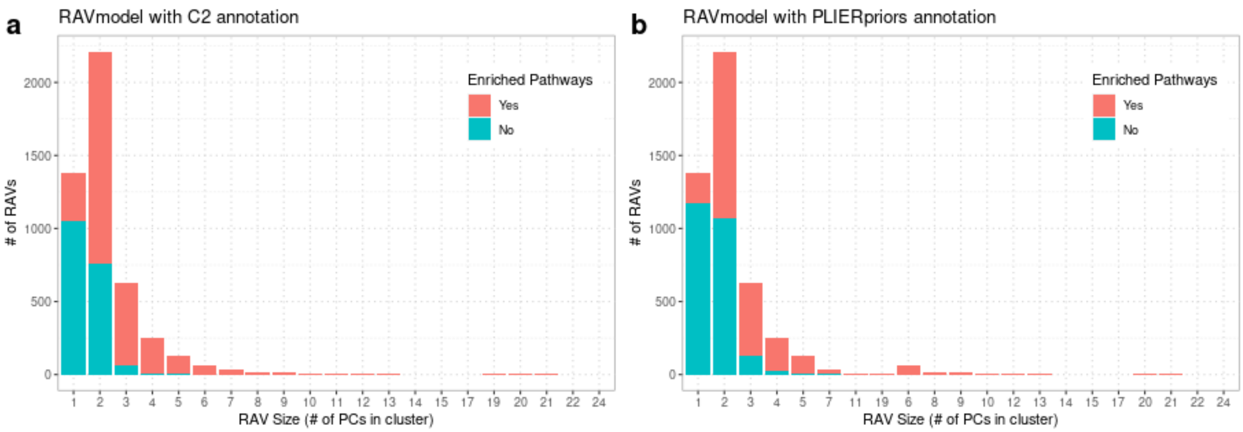

Supplementary Figure 6. Colon and rectal cancer associated RAV

Based on Fig. 2a, RAV832 seems to be associated with TCGA-COAD and TCGA-READ. Top validation results of **a** TCGA-COAD and **b** TCGA-READ include RAV832 with the negative average silhouette width. **c** MeSH terms associated with RAV832. **d** Studies contributing to RAV832. **e** MSigDB C2 gene sets enriched in RAV832.

a

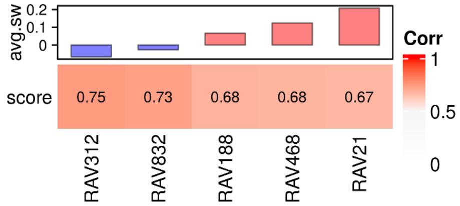

b

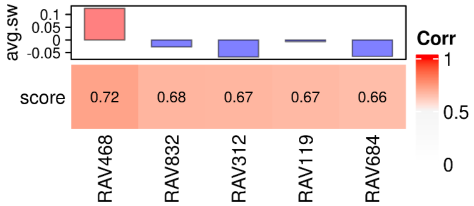

c

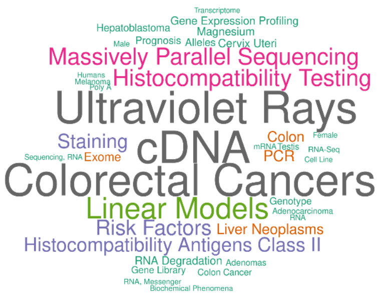

d

studyName	PC	Variance explained (%)	title
SRP010181	2	8.67	Derivation of HLA types from shotgun sequence datasets
SRP029880	1	20.16	Gene expression profiling study by RNA-seq in colorectal cancer
SRP068591	4	3.03	Gene signature in sessile serrated polyps identifies colon cancer subtype
SRP073267	9	2.16	Impact of RNA degradation on fusion detection by RNA-seq
SRP123604	9	0.66	Immune Profiling of Premalignant Lesions in Patients with Lynch Syndrome

e

RAV832.Description	RAV832.NES
SABATES_COLORECTAL_ADENOMA_UP	3.024180
HSIAO_LIVER_SPECIFIC_GENES	2.902520
GRADE_COLON_AND_RECTAL_CANCER_UP	2.815155
REACTOME_EUKARYOTIC_TRANSLATION_ELONGATION	2.685421
REACTOME_SRP_DEPENDENT_COTRANSLATIONAL_PROTEIN_TARGETING_TO_MEMBRANE	2.667746
KOBAYASHI_EGFR_SIGNALING_24HR_DN	2.647969
KEGG_RIBOSOME	2.642869
LEE_LIVER_CANCER_ACOX1_DN	2.635660
REACTOME_METABOLISM_OF_AMINO_ACIDS_AND_DERIVATIVES	2.601229
REACTOME_REGULATION_OF_EXPRESSION_OF_SLITS_AND_ROBOS	2.598015

Supplementary Figure 7. CRC characterization with different RAVs

In the Supplementary Note 3, we described two additional pairs of RAVs, RAV1575/834 and RAV188/832, that are potentially useful for CRC characterization. We applied the same analysis procedure on 18 CRC datasets as in Fig. 3 using those two pairs of RAVs. For the panel **a** and **d**, we assigned sample scores to 3,567 tumor samples from 18 CRC studies. The samples in each of 18 datasets, assigned to either (i) one of the 4 previously proposed CMS subtypes by CRC Subtyping Consortium or (ii) not assigned to a CMS subtype (so 5 x 18 = 90 total groups), are represented by the mean (point) and standard deviation (error bar) of sample scores. CMS subtypes (colors) separate when plotted in RAV coordinates. **(a-c)** CRC characterization with RAV1575/834. RAV1575 and RAV834 were identified based on their similarity to PCSS1 and PCSS2, respectively. **(d-f)** CRC characterization with RAV188/832. RAV188 and RAV832 were most frequently found among the top 10 validated RAVs (Supplementary Data 4). Boxplot statistics are summarized in Supplementary Data 5 and raw data are available in Supplementary Data 7.

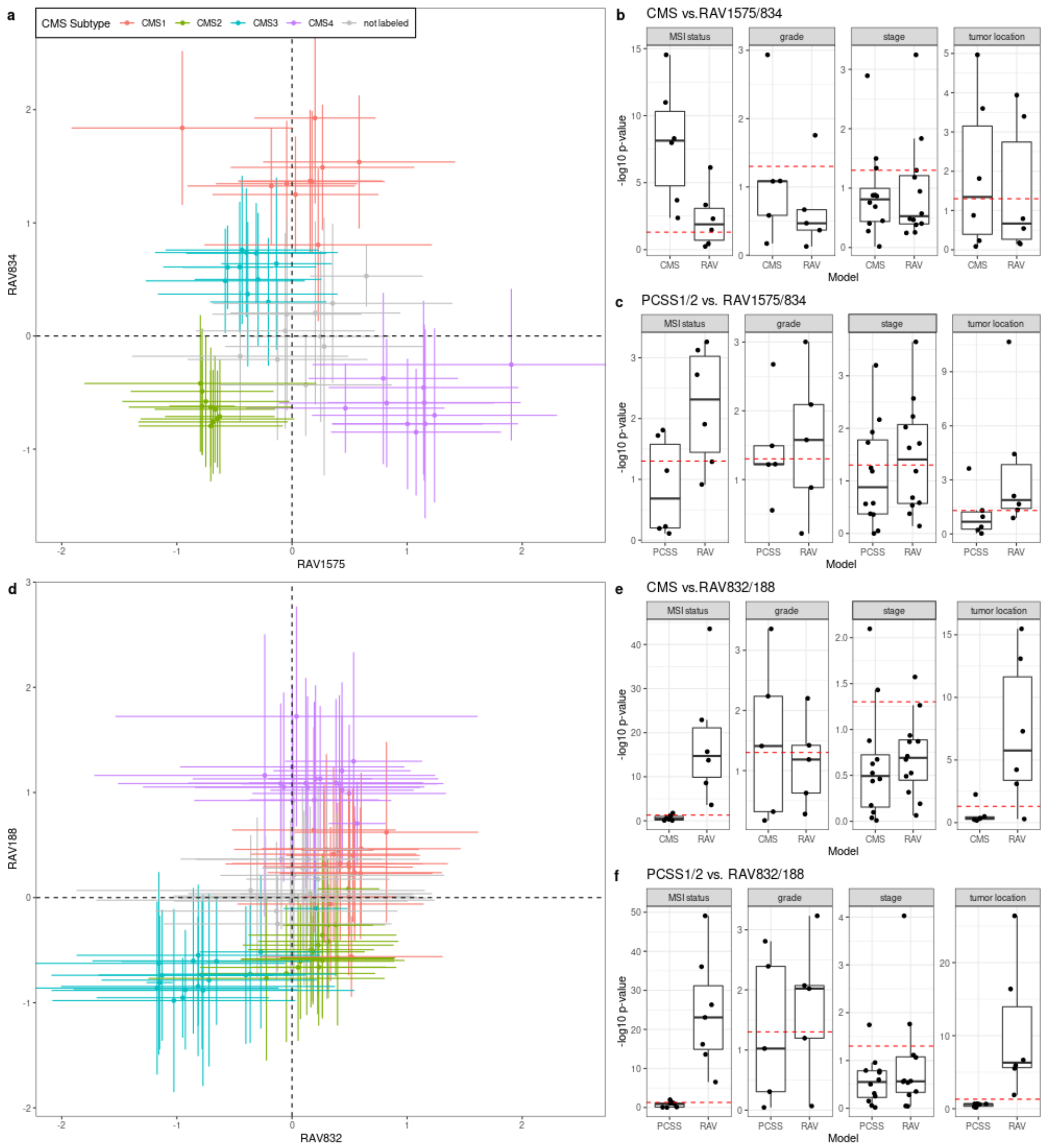

Supplementary Figure 8. CRC characterization with 10 validation datasets

Analyses in Fig. 3 were repeated with only 10 CRC datasets, excluding 8 datasets used to train PCSSs. **a** Subtype- and study-specific mean of PCSS1 and PCSS2 scores are plotted as points while the error bars represent standard deviation. **b** The same plotting scheme as the panel a was applied on RAV834 and RAV833-assigned scores. (**c-e)** LRTs compare the full model to a simplified model containing only **c** CMS subtypes or PCSS1/2, **d** CMS subtypes or RAV834/833, and **e** PCSS1/2 or RAV834/833. Boxplot statistics are summarized in Supplementary Data 5 and underlying data are included as Supplementary Data 8.

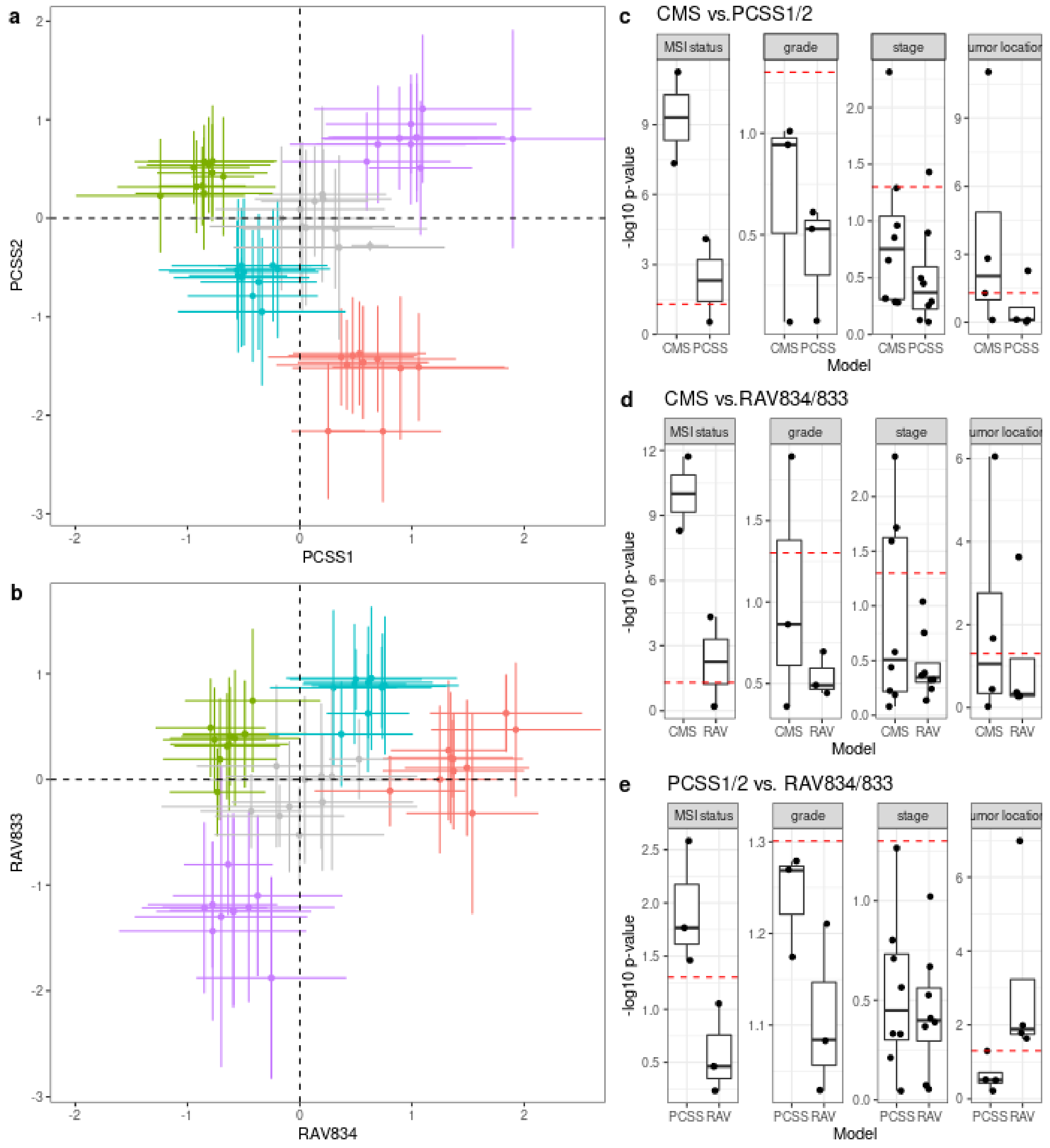

Supplementary Figure 9. Overview validation results via an interactive plot

In Fig. 2b, we used a table format to display the validation results. To understand the overall validation pattern for each PCs of new data, we provide an interactive plot as one of the visualization options. Here, we plotted the validation plot of the Human B-cell expression dataset (GSE2350) generated from the microarray. X-axis represents the average silhouette width and y-axis represents the validation score. Each point represents RAV, where the color shows the PC with the highest validation score for a given RAV. The point size reflects the cluster size, the number of PCs contributing to a given RAV. In general, we interpret that the points toward the upper right corner with the intermediate sizes are more relevant to new data than the others. An interactive form of this graph is available with the argument

`interactive=TRUE`, allowing the user to hover each data point for more information, such as cluster number and exact cluster size.

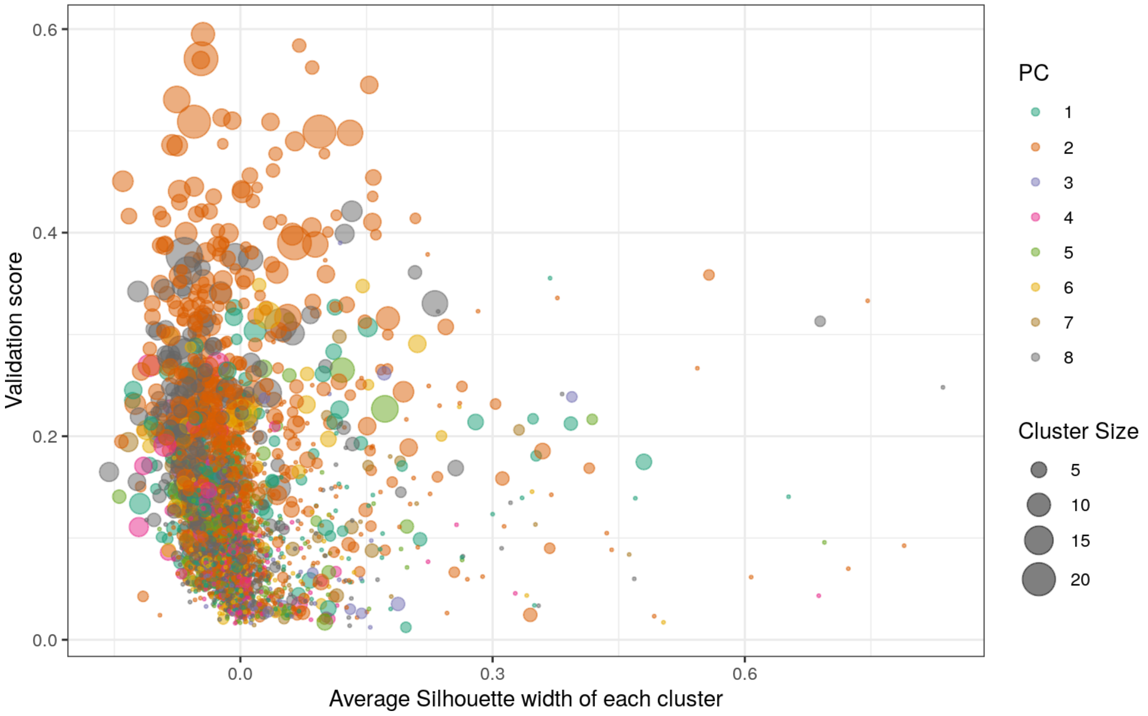

Supplementary Figure 10. PCA with GSEA annotation

PCA result of leukocyte gene expression data (E-MTAB-2452) is displayed in **(a)** a table or **(b)** a scatter plot. PCA is done on a centered, but not scaled, input dataset by default. Different cutoff parameters for GSEA annotation, such as minimum validation score or NES, can be set.

a

PC1.RAV23	PC2.RAV1552	PC3.RAV1387	PC4.RAV684
SVM T cells CD8	IRIS_Monocyte-Day0	MIPS_55S_RIBOSOME_MITOCHONDRIAL	REACTOME_CELL_CYCLE
SVM T cells CD4 naive	IRIS_DendriticCell-Control	REACTOME_RESPIRATORY_ELECTRON_TRANSPORT_ATP_S...	REACTOME_CELL_CYCLE_MITOTIC
SVM T cells follicular helper	DMAP_MONO2	MIPS_39S_RIBOSOMAL_SUBUNIT_MITOCHONDRIAL	NA
SVM T cells regulatory (Tregs)	IRIS_Monocyte-Day7	REACTOME_TCA_CYCLE_AND_RESPIRATORY_ELECTRON_T...	NA
SVM T cells gamma delta	SVM Monocytes	REACTOME_RESPIRATORY_ELECTRON_TRANSPORT	NA

b

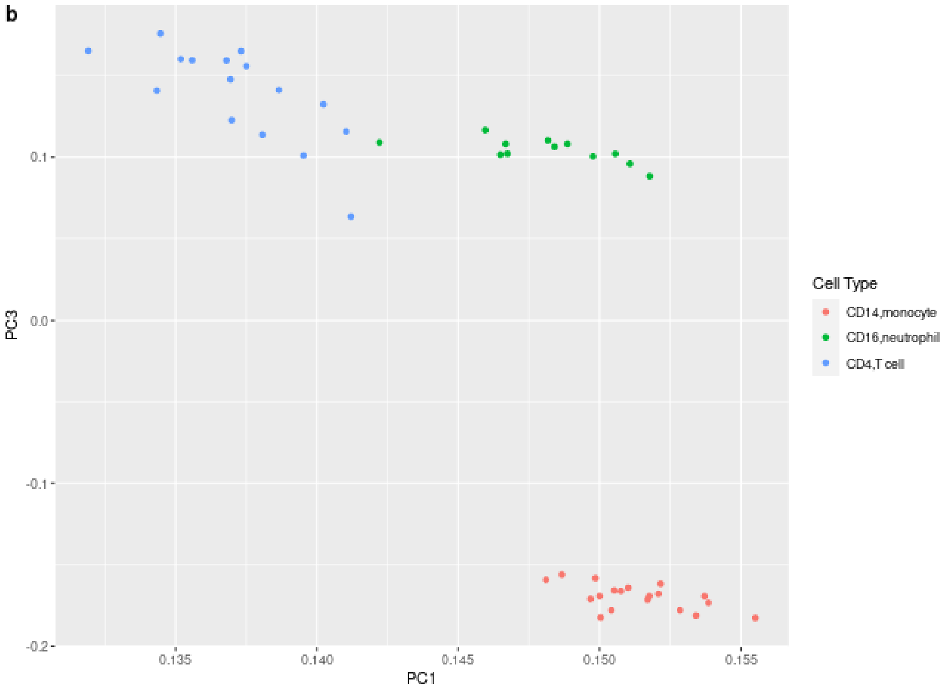

PC1.RAV23	PC3.RAV1387
SVM T cells CD8	MIPS_55S_RIBOSOME_MITOCHONDRIAL
SVM T cells CD4 naive	REACTOME_RESPIRATORY_ELECTRON_TRANSPORT_ATP_S...
SVM T cells follicular helper	MIPS_39S_RIBOSOMAL_SUBUNIT_MITOCHONDRIAL
SVM T cells regulatory (Tregs)	REACTOME_TCA_CYCLE_AND_RESPIRATORY_ELECTRON_T...
SVM T cells gamma delta	REACTOME_RESPIRATORY_ELECTRON_TRANSPORT

Supplementary Tables

Supplementary Table 1. Summary of new terms

Terms	Description

GenomicSuperSignature	Name of the R/Bioconductor package that contains all the functions to apply RAVmodel to new data, serving as a 'toolbox'. RAVmodels stored in Google Bucket are downloadable using the `getModel` function of the package.
GenomicSignatures-object	Data structure inherited from SummarizedExperiment
PCAGenomicSignatures-object	Data structure inherited from GenomicSignatures
RAV<br>(Replicable Axes of Variation)	A vector containing the average of loadings in each cluster.
RAVindex	A matrix containing all the RAVs. Rows are genes and columns are RAVs.
RAVmodel	PCAGenomicSignatures-object. It contains RAVindex, metadata on model building, and annotation. Different versions of RAVmodels are available.
Validation Score	The highest Pearson Correlation between top 8 PCs of new data and RAVs. Validation score provides a quantitative representation of the relevance between a new dataset and RAV. Process of comparing top PCs and RAVs is referred to as 'validation' and the RAV that gives the validation score is called 'validated RAV'.
Sample Score	The matrix multiplication result between the ‘samples x genes’ matrix of a new dataset and RAVindex. Similar to validation score, sample score provides a quantitative representation of the relevance between samples and the given RAV.

Supplementary Table 2. Available RAVmodels

This is the list of RAVmodels used in this work that are different in 1) the size of training datasets (‘studies’, ‘runs’, ‘samples’, and ‘genes’ columns), 2) the number of top PCs collected from each study (‘Top PCs’ column), 3) the number of clusters for hierarchical clustering (‘RAVs’ and ‘d’ columns), and 4) gene sets used for GSEA annotation (‘Genesets’ column). ‘Category’ column represents the main purpose of the RAVmodel, ‘Name’ column shows the specific name of RAVmodels, ‘Size (Mb)’ column tells the size of the given RAVmodel R object in a megabyte

unit. `RAVmodel_C2` and `RAVmodel_PLIERpriors`, are available for download using the `getModel` function and the others are available upon request.

Category	Name	Size (Mb)	studies	runs	samples	Top PCs	RAVs	d	genes	Genesets
Default RAVmodel	RAVmodel_C2	476	536	44890	34616	20	4764	2.25	13934	MSigDB C2 (ver.7.1)
Default RAVmodel	RAVmodel_PLIERpriors	475	536	44890	34616	20	4764	2.25	13934	PLIER priors (bloodCellMarkersIRISDMAP, canonicalPathways, and svmMarkers)
Model building variations	RAVmodel_C2_10PC	237	536	44890	34616	10	2382	2.25	13934	MSigDB C2 (ver.7.1)
Model building variations	RAVmodel_C2_clNum4	268	536	44890	34616	20	2680	4	13934	MSigDB C2 (ver.7.1)
Different size of training datasets	RAVmodel_1399	712	1399	75433	NA	NA	NA	NA	7951	MSigDB C2 (ver.7.1)

Supplementary Table 3. Comparison between GenomicSuperSignature and MultiPLIER

	**GenomicSuperSignature**	**MultiPLIER**
**Model name**	RAVmodel	recount2_MultiPLIER
**Model size**	~470Mb	2.1Gb<br>(a part of 81Gb tar.gz file stored in

		figshare)
**Model access**	Download using function	Download from flagshare
**Model availability**	RAVmodel_C2, RAVmodel_PLIERpriors	recount2_MultiPLIER
**Number of signatures**	4,764 RAVs	987 LVs
**Pathway Coverage for PLIER priors**	0.64	0.42
**Pathway Separation for PLIER priors**	Yes	Yes
**Projection on new data**	Functions from the package	Run scripts in GitHub repository
**Package**	GenomicSuperSignature R package	n.a.
**Training data**	44,890 runs from 536 studies	37,027 runs from 1,466 studies
**Annotation**	Literatures, MeSH terms, Gene sets	Gene sets
**Dimensional Reduction**	PCA and Clustering	PLIER
**Model building time**	~2 days	~2 weeks
**Recovering training data**	Yes	No
**Bioconductor Implementation**	Yes	No
**Galaxy web-tool Implementation**	Yes	No

Supplementary Table 4. Comparison between GenomicSuperSignature and Seurat's weighted nearest neighbor

	**GenomicSuperSignature**	**Seurat**
**Model**	RAVmodels	Human - PBMC reference atlas
**Data compression**	PCA and Clustering	Weighted nearest neighbor
**Annotation**	literatures, MeSH terms, gene sets	cell types

**# of samples used**	44,890 bulk RNAseq data	161,764 single cells (RNA and ADT data)
**# of datasets used**	536 heterogeneous, independent studies	8 volunteers for HIV vaccine at 3 time points before and after vaccine administration
**Source of datasets**	Public archives	Experiments by the authors
**Projection**	Pearson correlation between input's PCs and RAVs	Anchors through mutual nearest neighbor cells between reference and input's PCs from sPCA (supervised PCA)
**Recommended input**	RNAseq or microarray data with any underlying biology	scRNAseq data consisting of PBMC
**Transfer learning**	Any biological features RAVs represent	Immune cell types and states

Supplementary Notes

Supplementary Note 1. Comparison to existing tools

We compared GenomicSuperSignature with the two existing methods using large databases for transfer learning, MultiPLIER[4] and weighted nearest neighbor (WNN)[5] (Supplementary Table 3 and Supplementary Table 4). MultiPLIER is a transfer learning framework for rare disease study and its signal is named as latent variables (LVs) which are similar in concept to RAVs. We included a biological example inspired by MultiPLIER (Fig. 4). Our approach, while arriving at a similar result for this example, differs fundamentally from the approach applied in the MultiPLIER and offers distinct advantages. Whereas MultiPLIER identified the neutrophil-associated signal with the help of a relevant pre-established, single-dataset PLIER model, our method recovers this neutrophil signal in three unsupervised steps: validation of

dataset signals through matching to RAVs, keyword searching against enriched gene sets for RAVs, and neutrophil-associated metadata.

With respect to implementation, GenomicSuperSignature software and RAVmodels differ from MultiPLIER in their construction process. In addition, RAVmodel provides versatile functionalities including software tooling for directly indexing new data against the RAVs and annotation of principal components of new data. Below we list some of the novel aspects and differences between the RAVmodel and the MultiPLIER method and its implementation.

1) MultiPLIER uses a dimensional reduction method called PLIER. RAVmodel uses principal component analysis followed by clustering of similar PCs from large training datasets.
2) For MultiPLIER, the PLIER dimensional reduction process is constrained by gene sets, so different gene sets require independent models even though the training datasets are identical. However, because gene set annotation of RAVmodel is not a part of RAVindex building, a single RAVindex can be annotated with different gene sets. This modularity and flexibility is one of the strengths RAVmodel has over the existing tools.
3) Unlike LVs, RAVs maintain the information on which primary studies contribute to the signal: the publication, the PC, and the variance explained by the PC. This metadata enables the direct connection of new data to individual existing studies. As a result, any new knowledge and metadata associated with the training datasets can be immediately incorporated into the RAVmodel and used for new data analysis.
4) Software: RAVmodel is a component of our GenomicSuperSignature Bioconductor package and Galaxy web tool for easy application of the model on new data. However, MultiPLIER is solely a transfer learning model and no specific software is provided for its application.
5) Model availability: To obtain the recount2 MultiPLIER model, users need to download a 81Gb zipped file from figshare, which includes 2.1Gb of model. On the other hand, different versions of RAVmodels are stored in Google Cloud bucket and users can download < 500Mb RAVmodels using `wget` or the `getModel` function provided in the GenomicSuperSignature package. The RAVmodels themselves are formalized as a subclass of the ubiquitous SummarizedExperiment Bioconductor class and, as such, will feel familiar to Bioconductor users.
6) Database search: RAVmodel enables unsupervised and coherent database search of sample metadata, study data, and PCs from user data, whereas MultiPLIER does not have any database search capability.
7) Enhanced interpretability of PCA: RAVmodel links PCs of the input data with the most relevant RAVs, which subsequently links PCs to the existing database of studies, sample metadata, and pathway enrichment. This process of ‘labeling PC’ is implemented as the `annotatePC` and `plotAnnotatedPC` functions in the GenomicSuperSignature package. There is no comparable functionality in MultiPLIER.

WNN is developed for transfer learning of single-cell multimodal data and implemented in Seurat[5]. For information transfer purposes, a reference atlas from 161,764 cells was built from 8 volunteers for HIV vaccine before and after the vaccine administration, which is not public data.

These cells were thoroughly analyzed at the RNA and protein levels by the authors. Query data is linked to this reference via clustering (weighted-nearest neighbor) and the mutual nearest neighbor cells serve as 'anchors' to transfer information from the reference to query data. These algorithmic features and "training" data make Seurat's WNN applicable largely to input datasets consisting of or containing PBMC for identification of immune cell type and states. The training data for RAVmodel is instead 44,890 bulk RNAseq data from 536 independent studies available through public archives. Instead of providing a reference map like Seurat's WNN, our data compression procedure creates a data index, the RAVmodel, that connects literature, gene set annotation, sample metadata, and compressed gene expression signals. User-supplied, new query data can be linked to the RAVmodel through correlation between RAVs and query data's PCs. RAV transfers information across different databases and independent studies in an unsupervised manner.

Supplementary Note 2. Software implementation

The PCAGenomicSignatures class inherits SummarizedExperiment data structure and stores RAVindex, metadata, and annotation, which we collectively refer to as the RAVmodel (Supplementary Fig. 1b). Functions and S4 methods for the PCAGenomicSignatures class to access components of the RAVmodel, visualize analyses, and interpret new datasets are implemented in the GenomicSuperSignature R/Bioconductor package (Supplementary Table 1). We provide different versions of RAVmodels based on gene sets used for GSEA annotation and they are readily available (as .rds format files) to download from the internet through the `getModel` function or `wget` (Supplementary Table 2).

Two key functions, `validate` and `calculateScore`, allow interpretation of new datasets at the study level and at the individual sample level, respectively. The validate function calculates Pearson correlation coefficients between the top 8 PCs of a dataset and all RAVs (Supplementary Methods), from which the highest value is assigned as a 'validation score' of the corresponding RAV. Validation score provides a quantitative representation of the relevance between a new dataset and RAV. In general, the higher validation score implies that the RAV explains a more significant feature of a dataset. Validation outputs can be visualized as a heatmap table (Fig. 2a and 2b) and an interactive plot (Supplementary Fig. 9), through heatmapTable and plotValidate functions, respectively. Average silhouette width of each cluster is available as a reference for quality control and as an additional filtering option to find significant RAVs. The calculateScore function calculates a RAV-assigned 'sample score' to each sample, which is the matrix multiplication result (B^t, red) between the 'samples x genes' matrix (Y^t, grey) and RAVindex (Z, blue) (Supplementary Fig. 1c). Similar to validation score, sample score provides a quantitative representation of the relevance between samples and the given RAV.

In addition to the study-level validation scores and the sample scores acquired through gene expression profile, we can access the knowledge comprising GenomicSuperSignature through various entry points, such as metadata, MeSH term, and keywords, because RAVmodel maintains the link between RAV and it's source data. (Fig. 1b).

Supplementary Note 3. RAVs for colorectal cancer characterization

To evaluate the performance of RAVs compared to PCSSs, we searched for colon cancer associated RAVs in three different ways. First, we ran Kruskal-Wallis rank sum test between CMS subtypes and RAV-assigned scores. Two RAVs with the highest chi-square, RAV834 and RAV833, were selected for further testing. Second, we identified two RAVs, RAV1575 and RAV834, with the highest absolute Pearson correlation coefficients with PCSS1 and PCSS2, respectively (0.59 and 0.56). Last, we calculated validation scores for 18 colorectal cancer (CRC) datasets from curatedCRCData[8] and collected top 10 validated RAVs from each dataset. We summarized the frequency of different RAVs validating each dataset without any additional filtering criteria and selected the top 2 most frequently validated RAVs, RAV188 and RAV832, which were captured 14 and 10 times, respectively (Supplementary Data 5). In spite of the major difference in training datasets, RAV834/833 showed a comparable performance on colon cancer subtyping to PCSS1/2 (Fig. 3a). Notably, RAVs identified by CMS metadata (RAV834/833) performed better at CRC subtyping than the validated RAVs (RAV188/832), suggesting that the most prominent feature shared by 18 CRC datasets is not their disease subtypes (Fig.3a and Supplementary Fig. 10d).

Supplementary Note 4. PCA plot annotated with pre-calculated GSEA

One of the widely used exploratory data analysis methods is PCA because PCA plots can provide a quick overview of sample composition and distribution. We couple PCs from new data with GSEA annotation of RAVmodel and enable the instant interpretation of PCA results through the associated RAVs. We showed this example using a microarray dataset from isolated immune cells (E-MTAB-2452)[26] and RAVmodel_PLIERpriors (Supplementary Table 2). GenomicSuperSignature performs PCA on a centered but not scaled dataset and identifies the most similar RAV for each PC. GSEA annotation of these matched RAVs can be summarized in a table (Supplementary Fig. 10a, `annotatePC` function). Currently, any pair of top 8 PCs can be used to generate a PCA plot and GSEA annotation will be displayed as a linked table (Supplementary Fig. 10b, `plotAnnotatedPCA` function).

Supplementary Note 5. Example of RAV interpretation

We identified a neutrophil-associated RAV, RAV1551, using biological information including enriched gene sets and validation of dataset by matching to RAVs. Here, we provide additional examples of RAV interpretation selected by the structure of RAVindex itself. As a first example, RAV3133 is the only single-element cluster consisting of a PC1, which is derived from SRA study SRP100652 containing 100 samples. This study investigated the gene expression effect of disease associated polymorphisms in the endoplasmic reticulum aminopeptidase genes ERAP1 and ERAP2[6]. PC1 of this dataset explains 16.2% of the variance and has only one enriched pathway with a very low NES. Interestingly, this dataset is zero-inflated (99.9% of counts are 0) and all the RAVs consisting of PCs from SRP100652 are tagged with the message implemented in GenomicSuperSignature as an interpretation guide. The other example is RAV2285 with a high proportion of PC1, where 15 out of 17 PCs in this RAV are PC1s while it also contains PC2 and PC5. Except one PC1, all the PCs in this RAV are from single cell RNA

sequencing analysis (scRNAseq). The exception is PC1 from SRP116952, where RNA sequencing was performed on both total mRNA and polysome-associated mRNA[7].

Supplementary References

1. Ma, S. *et al.* Continuity of transcriptomes among colorectal cancer subtypes based on meta-analysis. *Genome Biol.* **19**, 142 (2018).
2. Guinney, J. *et al.* The consensus molecular subtypes of colorectal cancer. *Nat. Med.* **21**, 1350–1356 (2015).
3. OmicIDX. http://omicidx.cancerdatasci.org/.
4. Taroni, J. N. *et al.* MultiPLIER: A Transfer Learning Framework for Transcriptomics Reveals

Systemic Features of Rare Disease. *Cell Syst* **8**, 380–394.e4 (2019).

5. Hao, Y. *et al.* Integrated analysis of multimodal single-cell data. *Cell* **184**, 3573–3587.e29 (2021).
6. Hanson, A. L. *et al.* Genetic Variants in ERAP1 and ERAP2 Associated With Immune-Mediated Diseases Influence Protein Expression and the Isoform Profile. *Arthritis Rheumatol* **70**, 255–265 (2018).
7. Sandri, B. J. *et al.* Distinct Cancer-Promoting Stromal Gene Expression Depending on Lung Function. *Am. J. Respir. Crit. Care Med.* **200**, 348–358 (2019).
8. Parsana, P. & Riester, M. Waldron L. curatedCRCData: clinically annotated data for the colorectal Cancer transcriptome. Bioconductor.
