# Supplementary material for: GenomicSuperSignature facilitates interpretation of RNA-seq experiments through robust, efficient comparison to public databases
Source: Nat Commun. 2022 Jun 27;13:3695. doi: 10.1038/s41467-022-31411-3 (PMC9237024; doi:10.1038/s41467-022-31411-3)
Supplement: Supplementary file 2 — Editorial Assessment Report [file 41467_2022_31411_MOESM2_ESM.pdf]

nature portfolio

GUIDED OPEN ACCESS

nature methods   nature communications   communications biology

Contents of this report

- **Manuscript details**: overview of your manuscript and the editorial team.
- **Review synthesis**: summary of the reviewer reports provided by the editors.
- **Editorial recommendation**: personalised evaluation and recommendation from all 3 journals.
- **Annotated reviewer comments**: the referee reports with comments from the editors.
- **Open research evaluation**: advice for adhering to best reproducibility practices.

About the editorial process

Because you selected the **Nature Portfolio Guided Open Access option**, your manuscript was assessed for suitability in three of our titles publishing high-quality work across the spectrum of methods research: *Nature Methods, Nature Communications*, and *Communications Biology*. More information about Guided Open Access can be found here.

Collaborative editorial assessment

Your editorial team discussed the manuscript to determine its suitability for the Nature Portfolio Guided OA pilot. Our assessment of your manuscript takes into account several factors, including whether the work meets the **technical standard** of the Nature Portfolio and whether the findings are of **immediate significance** to the readership of at least one of the participating journals in the Nature Portfolio Guided Open Access methods cluster.

Peer review

Experts were asked to evaluate the following aspects of your manuscript:

- **Novelty** in comparison to prior publications;
- **Likely audience** of researchers in terms of broad fields of study and size;
- **Potential impact** of the study on the immediate or wider research field;
- **Evidence** for the claims and whether additional experiments or analyses could feasibly strengthen the evidence;
- **Methodological detail** and whether the manuscript is reproducible as written;
- Appropriateness of the literature review.

Editorial evaluation of reviews

Your editorial team discussed the potential suitability of your manuscript for each of the participating journals. They then discussed the revisions necessary in order for the work to be published, keeping each journal's specific editorial criteria in mind.

Journals in the Nature portfolio will support authors wishing to transfer their reviews and (where reviewers agree) the reviewers' identities to journals outside of Springer Nature.

If you have any questions about review portability, please contact our editorial office at guidedOA@nature.com.

Manuscript details

Tracking number	Submission date	Decision date
GUIDEDOA-21-00130	1 July 2021	Click or tap to enter a date.

**Title**	GenomicSuperSignature: interpretation of RNA-seq experiments through robust, efficient comparison to public databases	**Corresponding author**	Sean Davis<br>**Affiliation:** University of Colorado Anschutz School of Medicine
**Preprint information**	There is a preprint of this manuscript posted at **bioRxiv**.	**Peer review type**	Single-blind

Editorial assessment team

**Primary editor**	**Lin Tang**<br>**Home Journal:** *Nature Methods*, ORCID: 0000-0002-6050-0424<br>**Email:** lin.tang@nature.com
**Editorial team members**	**Doaa Megahed**, *Nature Communications*, ORCID: 0000-0002-3455-2992<br>**George Inglis**, *Communications Biology*, ORCID: 0000-0002-9069-5242
**About your primary editor**	Lin Tang obtained his Ph.D. in Computational Biology at the CAS-MPG Partner Institute for Computational Biology, Chinese Academy of Sciences, studying transcriptome evolution. He was an Associate Editor for *Nature Communications* beginning in November 2016, and joined *Nature Methods* as a Senior Editor in September 2019. He handles genomics and computational methods for the journal.

Editorial assessment and review synthesis

**Editor's summary and assessment**	Oh et al. develop GenomicSuperSignature, a computational method for interpreting transcriptomic datasets through comparison to public archives. They build a knowledge graph using annotated Replicable Axes of Variation (RAV) derived from public datasets to interpret new datasets in an efficient way. The method is demonstrated using several application examples.<br><br>The editors felt the topic of interpreting new RNA-seq datasets using public datasets interesting, but, in light of the reviewer comments, think the conceptual advances of the method may not be sufficient to meet the criteria for *Nature Methods*.<br><br>That said, the editors of Nature Communications and Communications Biology would be willing to consider a suitably revised version should the authors be willing to address the comments highlighted below.
**Editorial synthesis of reviews**	While our reviewers find this work of potential interest, a number of key concerns were raised, including novelty and comparison to existing approaches, method description and evaluation, biological insights and applications, as well as other conceptual, technical and presentation issues.<br><br>For reconsideration at *Nature Communications*, a revised manuscript should address the same points outlined by the editors of *Communications Biology*, plus the building of a web-based interface to facilitate attribute searches and/or a significant expansion of the method as suggested by Reviewer #2 to increase the method's resource value in light of the concerns regarding conceptual novelty pointed out by the reviewers. An acceptable expansion would also be applying the current method to another OMICS data type besides RNASeq data.<br><br>At a minimum, a revised manuscript for *Communications Biology* should include:<br>1. Appropriate benchmarking to an alternative method such as MultiPLIER, as suggested by Reviewer #1.<br>2. Further discussion of the RAV index and distinguishing features of GenomicSuperSignature, as outlined by Reviewers #1 and #3.<br>3. Justification of PC selection, as noted by Reviewer #3.<br>4. While we would encourage you to include the features suggested by Reviewer #2, this point would not be necessary for a revision at *Communications Biology*.

Editorial recommendation

nature methods	Revision not invited	After careful consideration of the paper and reviewers' reports, the *Nature Methods* editors think that the conceptual advance demonstrated is not sufficient for publication in the journal.
nature communications	Major Revisions with extension of the study	While we believe the method addresses an important issue that is still far from addressed, the reviewers point out the existence of conceptually very similar tools. Thus, we believe significant methodological expansion would enhance the resource value of the work.
communications biology	Major Revisions	Given Reviewer #1's concerns about the limited methodological advance from GenomicSuperSignature, we agree that it would be necessary to include additional benchmarking to an alternative method. It would also be necessary to elaborate on the RAV index, as suggested by Reviewer #3. While we would strongly encourage the authors to include the features suggested by Reviewer #2, these points would not be necessary for the scope of a revision.

Next Steps

Recommendation Summary

- Option 1: Extensive revision for *Nature Communications*.
- Option 2: Revise for consideration at *Communications Biology*

See the previous page for details. Note that Nature Methods has determined that they cannot consider a revised manuscript for editorial reasons.

Revision

If you would like to follow our recommendation, please upload the revised manuscript, along with your point-by-point response to the reviewers' reports and editorial advice **using the link provided in the decision letter**.

Revision checklist

- Cover letter, stating to which journal you are submitting
- Revised manuscript
- Point-by-point response to reviews
- Updated **Reporting Summary** and **Editorial Policy Checklist**
- Supplementary materials (if applicable)

Submission elsewhere

To a journal outside of Nature Portfolio

If you choose not to follow our recommendation and prefer to submit elsewhere, we can share the reviews with another journal outside of the Nature Portfolio if requested. You will need to request that the receiving journal office contacts us at guidedOA@nature.com. We have included editorial guidance below in the reviewer reports and open research evaluation to aid in revising the manuscript for publication elsewhere.

Annotated reviewer reports

The editors have included some additional comments on specific points raised by the reviewers below, to clarify requirements for publication in the recommended journal(s). However, please note that all points should be addressed in a revision, even if an editor has not specifically commented on them.

Reviewer #1	
**Reviewer #1**	This reviewer has not chosen to waive anonymity. The reviewer's identity can only be shared with representatives of an established journal editorial office.
**Reviewer #1 expertise**	Bioinformatics, omics data analysis
**Editor's comments about this review**	This reviewer has raised concerns on the novelty and comparison to existing approaches, method description and evaluation, as well as other conceptual, technical and presentation issues. In particular, a revised manuscript should address this reviewer's concerns regarding benchmarking to alternative methods, such as MultiPLIER, and better distinguish any advantages of GenomicSuperSignature.
**Reviewer #1 comments**	
**Overview**	GenomicSuperSignature: interpretation of RNA-seq experiments through robust, efficient comparison to public databases<br><br>The paper presents a Bioconductor package to link RNA-seq profiles to commonly occurring Principal Components (Replicable Axes of Variation (RAV)) derived from a compendium of previously analyzed gene expression experiments. This mapping can then be used to annotate the new RNA-seq samples with meta-data (MeSH terms) from the matching RNA-seq experiments. Additionally, the authors suggest that the RAV values are suitable for subtyping and classification.<br><br>The R implementation is well written and documented, and at times more helpful than the manuscript. The method is described more or less complete, with some minor ambiguity. The RAV space properties are not well described in the document.<br><br>Overall novelty is low, given existing tools that seem nearly identical to the proposed method. (e.g. multiPLIER)

Specific comments

#	Reviewer comment	Editorial comment
**1**	The model used in the paper contains 4764 RAV vectors. These are calculated from 13934 genes that share high variability from 536 RNA-seq experiments. As such, the data compression/dimensionality reduction is fairly minimal.	
**2**	The total number of Principal Components (PCs) considered are the top 20 PCs of the 536 experiments, resulting in 10720. The clustering and merging of similar PCs results in 4764 RAVs. By looking into the model retrieved from the R package, it becomes evident that 1378 RAVs are unique to a single experiment. As such, the name Replicable Axes of Variation seems misleading.	
**3**	RAVs that hae support from multiple experiments are generally PCs with high explained variance (PC1, PC2, PC3). This is most likely due to the normalization techniques applied by the proposed method (z-score across all samples). As such, the first couple of PCs encode cell type. For example, RAV184 only consists of PC1.	
**4**	In general, the publication does treat the set of RAV vectors as a black box. The properties of them should be discussed in much more detail.	**Please expand discussion on the RAV vectors, and, generally, elaborate on the distinguishing features of GenomicSuperSignature compared to existing alternatives. This is required for consideration at either *Nature Communications* or *Communications Biology.***
**5**	Using Principal Components to characterize sample properties is not novel. There is an abundance of publications using PC values rather than gene expression.<br>E.g., https://www.researchgate.net/profile/Luis-Diambra/publication/236166414_Dynamical_Analysis_of_Circadian_Gene_Expression/links/53dc21fd0cf2a76fb667b382/Dynamical-Analysis-of-Circadian-Gene-Expression.pdf	

	The example of classifying colorectal cancer samples using pairs of RAVs the proposed method identifies RAVs that are most associated with CMS subtypes. Given the total number of 4764 RAVs there are more than 10 million possible combinations. Given the number of possible feature combinations, it doesn't seem too surprising that the RAVs perform as well as CRC. The same approach would most likely work just as well on the gene level selecting two genes and as features.	
**6**	The paper states that no single-cell experiments were used. However, the filtering was not done correctly and there seem to be single cell studies in the trained model. E.g. SRP173388	**Please ensure that the text accurately reflects the underlying datasets, or update the analyses appropriately to account for inclusion of single cell data. This is required for consideration at either *Nature Communications* or *Communications Biology.***
**7**	The identification of RAVs associated with biological features is extremely similar to the multiPLIER method, the methods seem to only vary in details. And it is not quite clear to me what the difference between the two methods is. In fact, Figure 4 in this document seems to be near identical to the multiPLIER publication Figure 3. In both publications, Neutrophil count is associated with a latent variable (LV) or RAV and the results seem near identical.	**As mentioned above, it's important that a revised manuscript includes a fair comparison to existing methods, and that the conceptual differences compared to these methods are discussed in depth.**
**8**	When studying the RAV loadings it seems that non-coding genes seems to have much higher absolute values than protein coding genes.<br>E.g., rev(sort(rowMeans(abs(RAVindex(RAVmodel)))))[1:100]<br>This is somewhat surprising to me. This kind of property will affect enrichment analysis on the RAV vectors, since most gene sets in MSigDB are protein coding to my knowledge.	
**9**	The authors mention that gene sets with fewer than 10 genes are excluded. It is not clear whether the gene sets were first filtered against the 13934 genes that are used in the analysis.	**Please expand on the level of methodological detail provided to facilitate comprehension and reproducibility. Note that our journals do not impose word lengths on the Methods**

		**section.**
**10**	The reported gene sets such as in Figure 2 E do not show significance values. It would be important to show those.	
**11**	Calculating gene set enrichment on PC loadings is not novel and has been applied in the past on numerous publications.<br>E.g., https://biodatamining.biomedcentral.com/articles/10.1186/s13040-015-0059-z	
**12**	Very similar tools exist. Mainly multiPLIER employs near identical methodology. As mentioned before, Figure 4 in this document is near identical to Figure 3 in the multiPLIER paper, suggesting near identical performance on the same datasets.	**Please provide side-by-side comparisons to multiPLIER or similar existing alternative methods for further consideration in either *Nature Communications* or *Communications Biology*.**
**13**	Other efforts to harmonize gene expression datasets to transfer information exist. Especially in the single-cell field, algorithms were developed to combine RNA-seq samples from multiple experiments. e.g. seurat 4:<br>https://www.cell.com/cell/fulltext/S0092-8674(21)00583-3?_returnURL=https%3A//linkinghub.elsevier.com/retrieve/pii/S0092867421005833%3Fshowall=true	
**14**	Being able to integrate the large publicly available gene expression datasets currently being available is a very relevant problem in the field right now.	
**15**	The examples used to demonstrate the performance of the described method are somewhat anecdotal, and it is not clear whether the method will perform well for other cases. In the whole manuscript, only a handful of RAVs are actually used (less than 10) out of more than 4700.	
**16**	The document would benefit from a more broad benchmark that covers the whole spectrum of RAVs and their ability to quantify specific biological meaning.	
**17**	The data use and scripts are all accessible. Especially, the bioconductor package is very user-friendly.	

Reviewer #2	
**Reviewer #2**	This reviewer has not chosen to waive anonymity. The reviewer’s identity can only be shared with representatives of an established journal editorial office.
**Reviewer #2 expertise**	Bioinformatics, omics data analysis
**Editor’s comments about this review**	This reviewer is positive about the work and has provided suggestions to strengthen the method.

Reviewer #2 comments	
**Overview**	The metadata morass problem is as follows. Investigators submit genomic data to public archives like the Sequence Read Archive (SRA), and: (1) they all use different words to describe their samples, so attributes do not have controlled vocabularies, and (2) they label their samples incompletely, so samples end up having missing attributes. Both (1) and (2) make it hard for folks to repurpose genomic data for new analyses, and this translates to money wasted on new experiments and insights lost because, e.g., high-value samples -- of rare disease states, tissues that are hard to access, organisms that no longer exist -- can't be identified and collected across studies to boost power, sometimes critical to make new analyses feasible.<br><br>Oh et al. have developed and written up a creative approach to solving the metadata morass. It's simple, it's versatile, and it's deployed at scale. The simple: they found what they call RAVs, or replicable axes of variation. After doing PCA on each sample's transcript quantifications across samples, PCs in samples with similar attributes cluster. So the authors performed hierarchical clustering of the top 20 PCs, with PCs in each cluster averaged to obtain centroids. Then they annotated centroids with enriched pathways from the Molecular Signatures Database (MSigDB) found via Gene Set Enrichment Analyses (GSEA) as well as with Medical Subject Headings (MeSH) terms found via bag-of-words on available metadata. The versatile: investigators now have new suggested annotations of old archived samples, and if they've collected new samples, these can now be annotated rapidly with gene sets and MeSH terms using the authors' GenomicSuperSignature R/Bioconductor package. The authors demonstrated utility across a diverse application space, showing one RAV correlated well with neutrophil count, and RAVs could distinguish colorectal cancer subtypes. The scale: nearly 45K publicly available RNA-seq samples indexed by refine.bio.

Specific comments

#	Reviewer comment	Editorial comment
1	Are the conclusions novel? Yes. Have the authors appropriately credited previously work? Mostly. Some people have done similarity search for single-cell stuff -- check out, e.g., https://academic.oup.com/nar/article/46/W1/W141/5000022 and https://www.nature.com/articles/s41592-021-01076-9.	**As pointed out by Reviewer 1, there's a clear need to not only discuss the conceptual novelty of the work but also benchmark their method against existing methods when appropriate.**
2	This paper should be published in *Nature Methods*. It will influence thinking in the field if it's advertised.<br><br>A web interface for attribute search could increase impact, maybe as part of refine.bio.	**While we appreciate the reviewer's input, we must emphasize that any decisions regarding publication are made by editors.**<br><br>**Given the concerns regarding novelty, building a web interface will significantly improve the resource value of the work. This is strongly encouraged, especially for *Nature Communications.***
3	The work is convincing.<br><br>**Suggestion for the authors:** it's possible you can do still better, at least according to this reviewer. Think feature hashing and variants from Kane and Nelson's https://arxiv.org/abs/1012.1577, and similarity search using out-of-box nearest-neighbor search tools. You can do dimensionality reduction on the fly, probably even in one pass by hashing, e.g., 32mers directly from the FASTQs into like 5000 bins, and then do PCA. Quantifying genes in between may be limiting. Not every sequence in your samples is going to be the organism reported; there'll be bacterial and viral sequences. Those could be RAVs, and the people want those RAVs, too. (Could you still do the GSEA? Maybe! There could be a strategy for obtaining enriched sequences from clusters, and then aligning those to the genome.) Stream those reads into a single-threaded process on a lonely machine in some	**To increase the resource value of the method, expanding the method either through a web-interface and/or the addition of more features would be necessary for further consideration at *Nature Communications.***<br><br>**While the editors at *Communications Biology* would strongly encourage you to include these features, this point would not be necessary for a revision.**

	data center for a year, and see what happens.<br>I do *not* think the suggestion above need be taken.	
**4**	The authors have provided the GitHub repo https://github.com/shbrief/GenomicSuperSignaturePaper with vignettes guiding the user through reproducing analyses. Necessary data are available for download from Google buckets free of charge. It's above and beyond.	

Reviewer #3

**Reviewer #3**	This reviewer has not chosen to waive anonymity. The reviewer's identity can only be shared with representatives of an established journal editorial office.
**Reviewer #3 expertise**	Bioinformatics, omics data analysis
**Editor's comments about this review**	This reviewer has raised concerns on the method description and evaluation, biological insights and applications, as well as other conceptual, technical and presentation issues

Reviewer #3 comments

**Overview**	Oh et al develop the GenomicSuperSignature R/Bioconductor package for transfer learning using Replicable Axes of Variation (RAV), or gene signatures learned from ensembles of PCA loadings from 536 studies comprising 44,890 RNA sequencing profiles. They annotate these RAVs using the metadata of the original studies and additional gene set enrichment analysis. They demonstrate the ability of the RAVindx signatures to capture and transfer biological knowledge in ways that outperform current methods. Specifically, they find RAVs that are more closely related to colorectal carcinoma (CRC) clinicopathological variables than transcriptome subtypes previously identified through intensive analysis of CRC-specific databases bespoke subtyping efforts. They then identified an RAV that was highly correlated to neutrophil content using a systemic lupus erythematosus (SLE) dataset not included in the model training data, and used this RAV to estimate neutrophil content in a nasal brushing (NARES) dataset that lacks neutrophil count information. In all, the GenomicSuperSignature tool is a robust approach that enables analysis of new gene expression data in the context of existing databases using minimal computing resources.<br>While aware that RAVindex represents one of their key innovations, the authors fail

	to give the index itself the attention and description that it deserves in the manuscript. Instead, they focus on its application and stress what that then enables. This is understandable given the desire to present the utility of the tool, but the impact of the publication and the author's work suffers as a result. The amount of work to compile and annotated the RAVindex is by no means trivial. Moreover, the act of assembling and condensing the information contained in 44,890 RNA sequencing profiles should provide insights into trends in and features of the biological processes they are cataloging. Yet beyond claiming that GenomicSuperSignature establishes a knowledge graph, these insights are not elucidated. As the tool itself is robust and the manuscript a sufficient characterization of its utility, the manuscript merits publication in Nature Communications with a brief consideration of the follow. However, substantial effort to address the following issues (enumerated under "Strength of the claims") has the potential to greatly increase the impact of the publication and depending on the findings may merit reconsideration for publication *in Nature Methods*.<br><br>As the tool itself is accessible and robust, the code available and annotated, and the manuscript a sufficient characterization of its utility. In conjunction with the comments on impact addressing the follow would benefit the work.

Specific comments

#	Reviewer comment	Editorial comment
**1**	There is no global description of the RAV index beyond how it was assembled. The number of RAVs is not revealed until the discussion whereas this should be a result. What were the average cluster size that was condensed into a single RAV? What was the range and variation of cluster size? Summary statistics characterizing the RAVindx and biologically relevant features of its compilation should be presented in a subsection of the results.	**For the sake of reproducibility, please expand on these metrics. Please also refer to the Open Research Evaluation for other requirements regarding reproducibility, for further consideration at *Nature Communications* or *Communications Biology*.**
**2**	The claim that GenomicSuperSignature establishes a knowledge graph is not well substantiated.	
**3**	The selection of the top 20 PCs from each study seems arbitrary and dismissive of the potential to have a wide range of the number of informative PCs depending on the complexity and information content of a study. Methods for choosing the optimal number of PCs for a given study exist, i.e. the elbow method, etc., and should be assessed to justify the thresholding. I'm surprised that this wasn't	**This point would be necessary for further consideration at either journal.**

	pointed out by authors on review as their own work as implicated the necessity of considering multiple dimensionalizations in unsupervised learning as being necessary to capture varying levels of biological hierarchy.	

Reproducibility is also addressed in the "Strength of claim section". Additional minor issues to be addressed follow.

**4**	On Line 102 the sentence states: “Also, these tools either do not provide transfer learning from large public databases, or in the case of MultiPLIER, require substantial computing resources and bioinformatics expertise.” However, it would be more accurate to state that these methods “do not provide a reference catalog for transfer learning from large public databases”. This also accurately highlights contribution of this paper in providing this catalog.	
**5**	The claim that “VST transformation17 was excluded because it requires significantly more computing resources without any meaningful improvement on capturing biological signatures over log2-transformation.” Needs to either be substantiated via a quantification or a citation.	
**6**	Using a varying number of PCs would add a complexity to the process that seemed unjustified given that the variance explained by each PC does not vary much by study size. This needs to be quantified.	
**7**	ICA can be considered a reordering of PCA and is thus derivative. “We also ruled out independent component analysis (ICA) because it assumes that subcomponents are independent to each other, which we considered not the case for biological data.”	
**8**	Line 481 “Also, low- or non-expressing genes can be rather a noise, making it harder to interpret the result.” is grammatically incorrect.	
**9**	Line 568 “The PCAGenomicSignatures class inherits SummarizedExperiment data structure and stores RAVindex, metadata, and annotation, which we collectively refer to as the RAVmodel (Supplementary Fig. 1B).” has an extra space.	

Open research evaluation

Data availability

Data Availability statement

Please add a Data Availability statement. Please ensure that your Data Availability statement includes accession details for deposited data, mentions where Source data can be found, and states that all other data are available from the corresponding author (or other sources, as applicable) on reasonable request. More information about our data availability policy can be found here: https://www.nature.com/nature-portfolio/editorial-policies/reporting-standards#availability-of-data

See here for more information about formatting your Data Availability Statement: http://www.springernature.com/gp/authors/research-data-policy/data-availability-statements/12330880

Nature Portfolio journals strongly support public availability of data and custom code associated with the paper in a persistent repository where they can be freely and enduringly accessed or as a supplementary data file when no appropriate repository is available. If data and code can only be shared on request, please explain why in your data Availability Statement, and also in the correspondence with your editor. For more information, please refer to https://www.nature.com/nature-research/editorial-policies/reporting-standards#availability-of-data

Please ensure that datasets deposited in public repositories are now publicly accessible, and that accession codes or DOI are provided in the "Data Availability" section. As long as these datasets are not public, we cannot proceed with the acceptance of your paper. For data that have been obtained from publicly available sources, please provide a URL and the specific data product name in the data availability statement. Data with a DOI should be further cited in the methods reference section.

Data citation

Please cite (within the main reference list) any datasets stored in external repositories that are mentioned within their manuscript. For previously published datasets, we ask that you cite both the related research article(s) and the datasets themselves. For more information on how to cite datasets in submitted manuscripts, please see our data availability statements and data citations policy: https://www.nature.com/documents/nr-data-availability-statements-data-citations.pdf

Citing and referencing data in publications supports reproducible research, by increasing the transparency and provenance tracking of data generated or analysed during research. Citing data formally in reference lists also helps facilitate the tracking of data reuse and may help assign credit for individuals' contributions to research. A number of Springer Nature imprints are signatories of the Joint Declaration on Data Citation Principles, which stress the importance of data resources in scientific communication.

Code availability and citation

Please include a statement under the heading "Code Availability", indicating whether and how the custom code/software reported in your study can be accessed, including any restrictions to access. This section should also include information on the versions of any software used, if relevant, and any specific variables or parameters used to generate, test, or process the current dataset. Code availability statements should be provided as a separate section after the Data Availability section.

Upon publication, Nature Portfolio journals consider it best practice to release custom computer code in a way that allows readers to repeat the published results. Code should be deposited in a DOI-minting repository such as Zenodo, Gigantum or Code Ocean and cited in the reference list following the guidelines described in our policy pages (see link below). Authors are encouraged to manage subsequent code versions and to use a license approved by the open source initiative.

Full details about how the code can be accessed and any restrictions must be described in the Code Availability statement.

See here for more information about our code availability policies: https://www.nature.com/nature-portfolio/editorial-policies/reporting-standards#availability-of-computer-code

We also provide a Code and Software submission checklist that you may find useful: https://www.nature.com/documents/nr-software-policy.pdf

Please note: because of advanced features used in this form, you must use Adobe Reader to open the documents and fill it out.

Ethics

Please provide a 'Competing interests' statement using one of the following standard sentences:

1. The authors declare the following competing interests: [specify competing interests]
2. The authors declare no competing interests.

See our competing interests policy for further information: https://www.nature.com/nature-research/editorial-policies/competing-interests

Reporting and reproducibility

Reporting

All data that support the conclusions drawn must be presented in the manuscript unless they are published elsewhere. Nature Portfolio journals do not allow statements of “data not shown”.

Reproducibility

Please state in the legends how many times each experiment was repeated independently with similar results. This is needed for all experiments, but is particularly important wherever results from representative experiments (such as micrographs) are shown. If space in the legends is limiting, this information can be included in a section titled “Statistics and Reproducibility” in the methods section.

Statistics

Wherever statistics have been derived (e.g. error bars, box plots, statistical significance) the legend needs to provide and define the n number (i.e. the sample size used to derive statistics) as a precise value (not a range), using the wording “n=X biologically independent samples/animals/cells/independent experiments/n= X cells examined over Y independent experiments” etc. as applicable.

Statistics such as error bars, significance and p values cannot be derived from n<3 and must be removed from all such cases.

We strongly discourage deriving statistics from technical replicates, unless there is a clear scientific justification for why providing this information is important. Conflating technical and biological variability, e.g., by pooling technically replicates samples across independent experiments is strongly discouraged. (For examples of expected description of statistics in figure legends, please see the following https://www.nature.com/articles/s41467-019-11636-5 or https://www.nature.com/articles/s41467-019-11510-4).

All error bars need to be defined in the legends (e.g. SD, SEM) together with a measure of centre (e.g. mean, median). For example, the legends should state something along the lines of "Data are presented as mean values +/- SEM" as appropriate.

All box plots need to be defined in the legends in terms of minima, maxima, centre, bounds of box and whiskers and percentile.

Legends requiring revision:

1. Please note that the error bars need to be defined in the legends of supplementary figures 5a, d.
2. If the shaded areas denote error bands then the error bands need to be defined in the legends of figures 4a-c.

Please note that the box plots need to be defined in terms of minima, maxima, centre, bounds of box and whiskers and percentile in the legends of figures 3b, c and supplementary figures 5b, c, e, f; 6c-e.

The figure legends must indicate the statistical test used. Where appropriate, please indicate in the figure legends whether the statistical tests were one-sided or two-sided and whether adjustments were made for multiple comparisons.

For null hypothesis testing, please indicate the test statistic (e.g. F, t, r) with confidence intervals, effect sizes, degrees of freedom and P values noted.

Please provide the test results (e.g. P values) as exact values whenever possible and with confidence intervals noted.

Data presentation

Please ensure that data presented in a plot, chart or other visual representation format shows data distribution clearly (e.g. dot plots, box-and-whisker plots). When using bar charts, please overlay the corresponding data points (as dot plots) whenever possible and always for $n \leq 10$. (Please see the following editorial for the rationale behind this request and an example https://www.nature.com/articles/s41551-017-0079).

Other notes

We have included as an attachment to the decision letter a version of your Reporting Summary with a few notes. This is mainly for your information, but we hope it is helpful when preparing your revised manuscript. If you decide to resubmit the manuscript for further consideration, please be sure to include an updated Reporting Summary.

Please note that the GitHub web-link provided for supplementary table 2 is not accessible. Please provide a valid and appropriate GitHub web-link.
