# Supplementary material for: GenomicSuperSignature facilitates interpretation of RNA-seq experiments through robust, efficient comparison to public databases
Source: Nat Commun. 2022 Jun 27;13:3695. doi: 10.1038/s41467-022-31411-3 (PMC9237024; doi:10.1038/s41467-022-31411-3)
Supplement: Supplementary file 3 — Description of Supplementary Data [file 41467_2022_31411_MOESM3_ESM.pdf]

Supplemental Data Descriptions

Supplementary Data 1. Training datasets used in this study

The study accession, the number of samples, and the title of 536 training datasets used to construct the current version of RAVmodel (`RAVmodel_536` column). The number of samples for each study based on the metadata (`metadata` column) and the number of samples actually used (`downloaded` column) are different due to the data availability at the time of download.

Supplementary Data 2. Source types of training datasets

We obtained the source name for 435 studies (~81.2% of all training datasets) from OmicIDX[3] and did a manual curation based on the source name (`source_name` column) to understand the types of training datasets. Curation covered four categories: 1) whether the dataset is cancer or not (`cancer` column), 2) whether the dataset is blood or not (`blood` column), 3) whether the dataset is cell line or not (`cell_line` column), 4) what is the origin of samples (`origin` column).

Supplementary Data 3. Distribution of PCs in different sized RAVs

We summarize the distribution of PCs in different sizes of RAVs. There are 21 different sizes of RAVs with the smallest containing a single PC while the largest containing 24 PCs. We collected all the PCs contributing to the given size of the clusters and plotted 21 barplots. We observed that one- and two-element clusters are predominantly from lower PCs. From three-element clusters, however, the skewness changes from left to right.

Supplementary Data 4. Summary of top 10 validated RAVs for 18 colorectal cancer datasets

Supplementary Data 5. Summary of boxplot statistics

This is the summary statistics of all the boxplots present in this work. It contains 72 rows from 9 boxplots (Fig.3b-c, Supplementary Fig.7b-c, Supplementary Fig.7e-f, and Supplementary Fig.8c-e), where each has 4 panels with 2 groups. It has 11 columns labeled as figure, panel, group, dataset_used (the number of datasets used that are represented as dots in the boxplots), minima, whisker_min, first_quartile, median, third_quartile, whister_max, and maxima. Four panel numbers denote MSI status (1), grade (2), stage (3), and tumor location (4). For groups, 1 and 2 represent left and right groups, respectively, in each panel.

Supplementary Data 6. Raw data for Figure 3

CRC characterization using CMS subtypes, PCSS1/2, and RAV834/833, demonstrated with 18 validation datasets.

Supplementary Data 7. Raw data for Supplementary Figure 7

CRC characterization with different RAVs using 18 validation datasets

Supplementary Data 8. Raw data for Supplementary Figure 8

CRC characterization using CMS subtypes, PCSS1/2, and RAV834/833, demonstrated with 10 validation datasets.
