# Supplementary material for: GenomicSuperSignature facilitates interpretation of RNA-seq experiments through robust, efficient comparison to public databases
Source: Nat Commun. 2022 Jun 27;13:3695. doi: 10.1038/s41467-022-31411-3 (PMC9237024; doi:10.1038/s41467-022-31411-3)
Supplement: Supplementary file 6 — Supplementary Dataset 3 [file 41467_2022_31411_MOESM6_ESM.pdf]

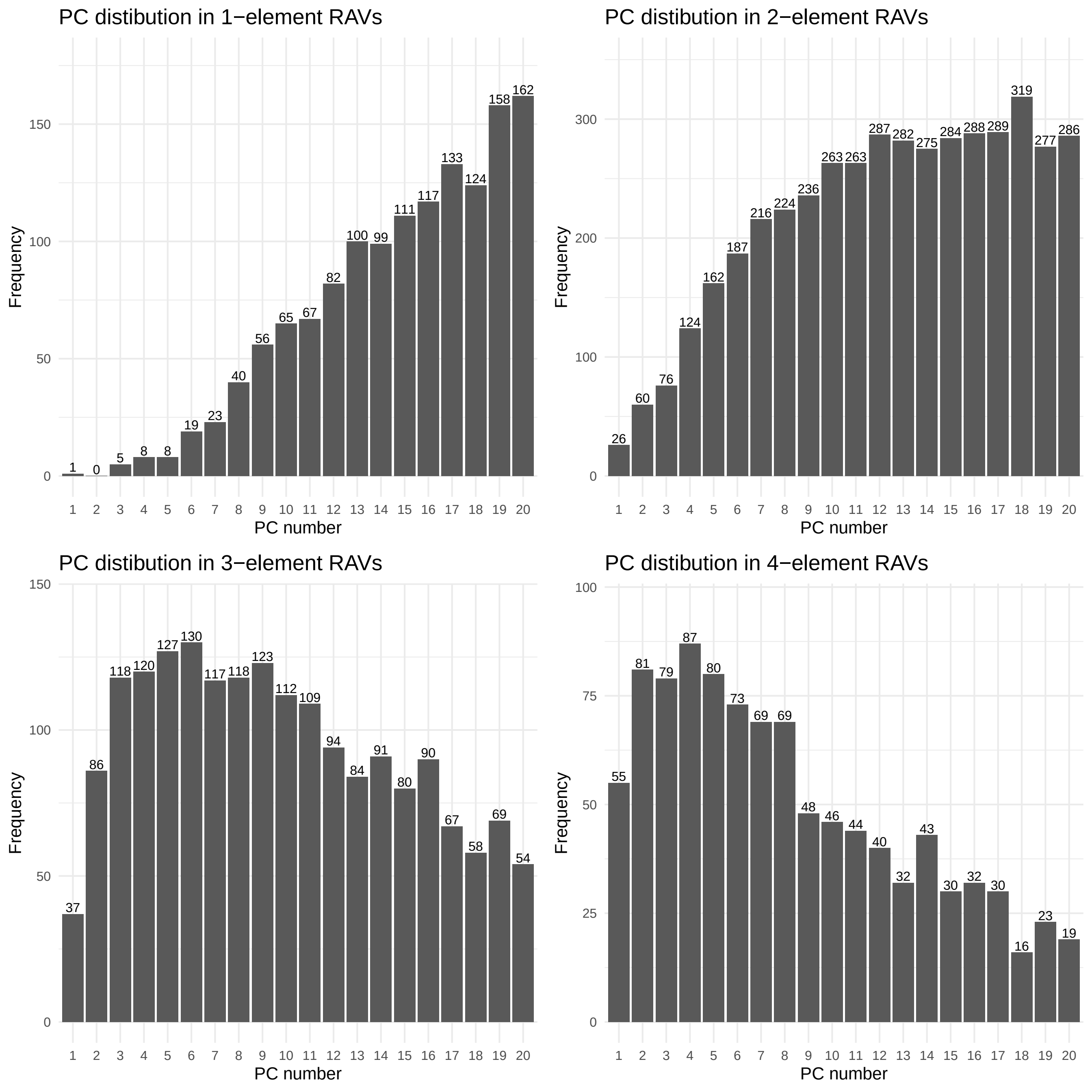
PC distibution in 1−element RAVs
Frequency
PC number
0
50
100
150
1 2 3 4 5 6 7 8 9 10 11 12 13 14 15 16 17 18 19 20
1 0 5 8 8 19 23 40 56 65 67 82 100 99 111 117 133 124 158 162
PC distibution in 2−element RAVs
Frequency
PC number
0
100
200
300
1 2 3 4 5 6 7 8 9 10 11 12 13 14 15 16 17 18 19 20
26 60 76 124 162 187 216 224 236 263 263 287 282 275 284 288 289 319 277 286
PC distibution in 3−element RAVs
Frequency
PC number
0
50
100
150
1 2 3 4 5 6 7 8 9 10 11 12 13 14 15 16 17 18 19 20
37 86 118 120 127 130 117 118 123 112 109 94 84 91 80 90 67 58 69 54
PC distibution in 4−element RAVs
Frequency
PC number
0
25
50
75
100
1 2 3 4 5 6 7 8 9 10 11 12 13 14 15 16 17 18 19 20
55 81 79 87 80 73 69 69 48 46 44 40 32 43 30 32 30 16 23 19

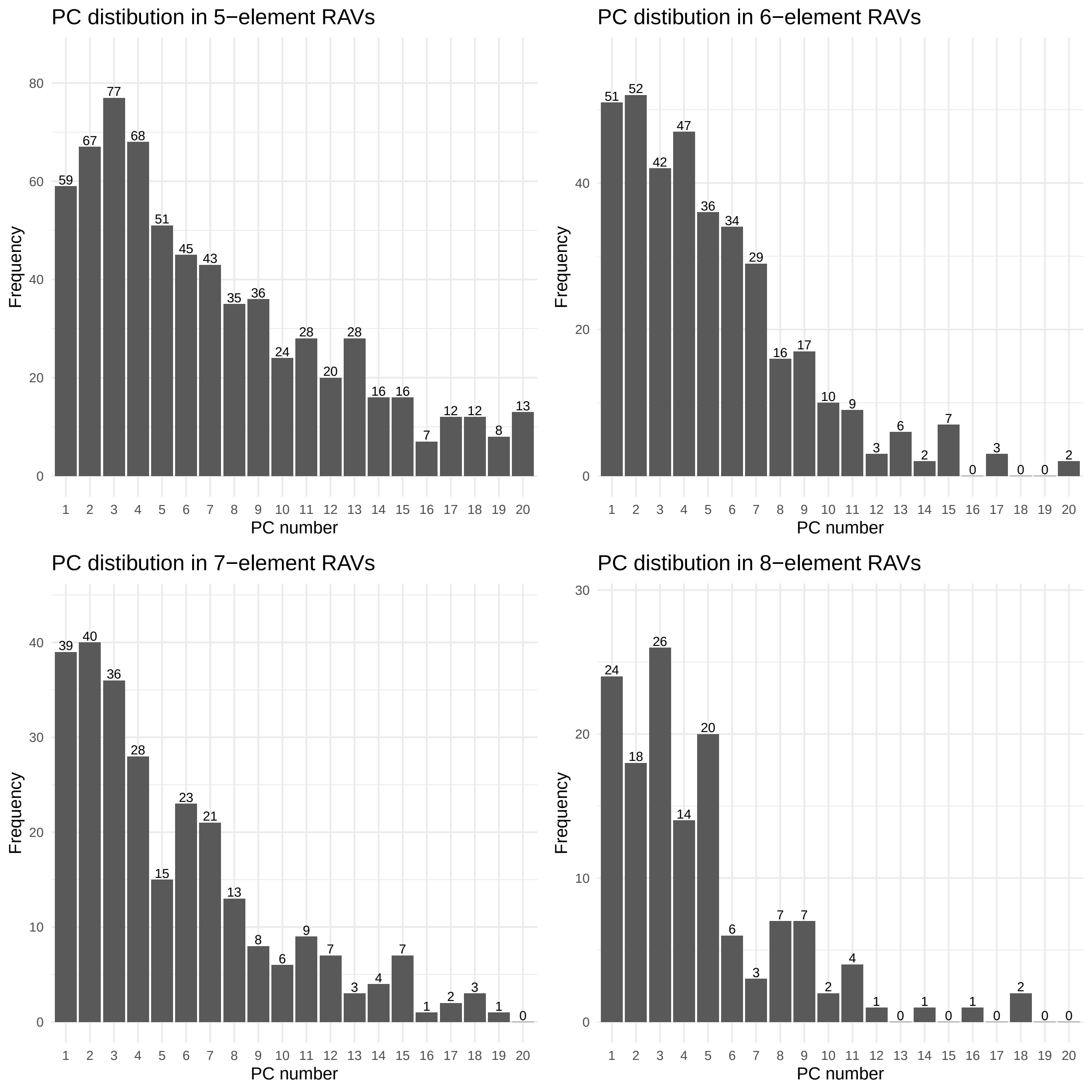
PC distibution in 5−element RAVs
Frequency
PC number
59 67 77 68 51 45 43 35 36 24 28 20 28 16 16 7 12 12 8 13
PC distibution in 6−element RAVs
Frequency
PC number
51 52 42 47 36 34 29 16 17 10 9 3 6 2 7 0 3 0 0 2
PC distibution in 7−element RAVs
Frequency
PC number
39 40 36 28 15 23 21 13 8 6 9 7 3 4 7 1 2 3 1 0
PC distibution in 8−element RAVs
Frequency
PC number
24 18 26 14 20 6 3 7 7 2 4 1 0 1 0 1 0 2 0 0

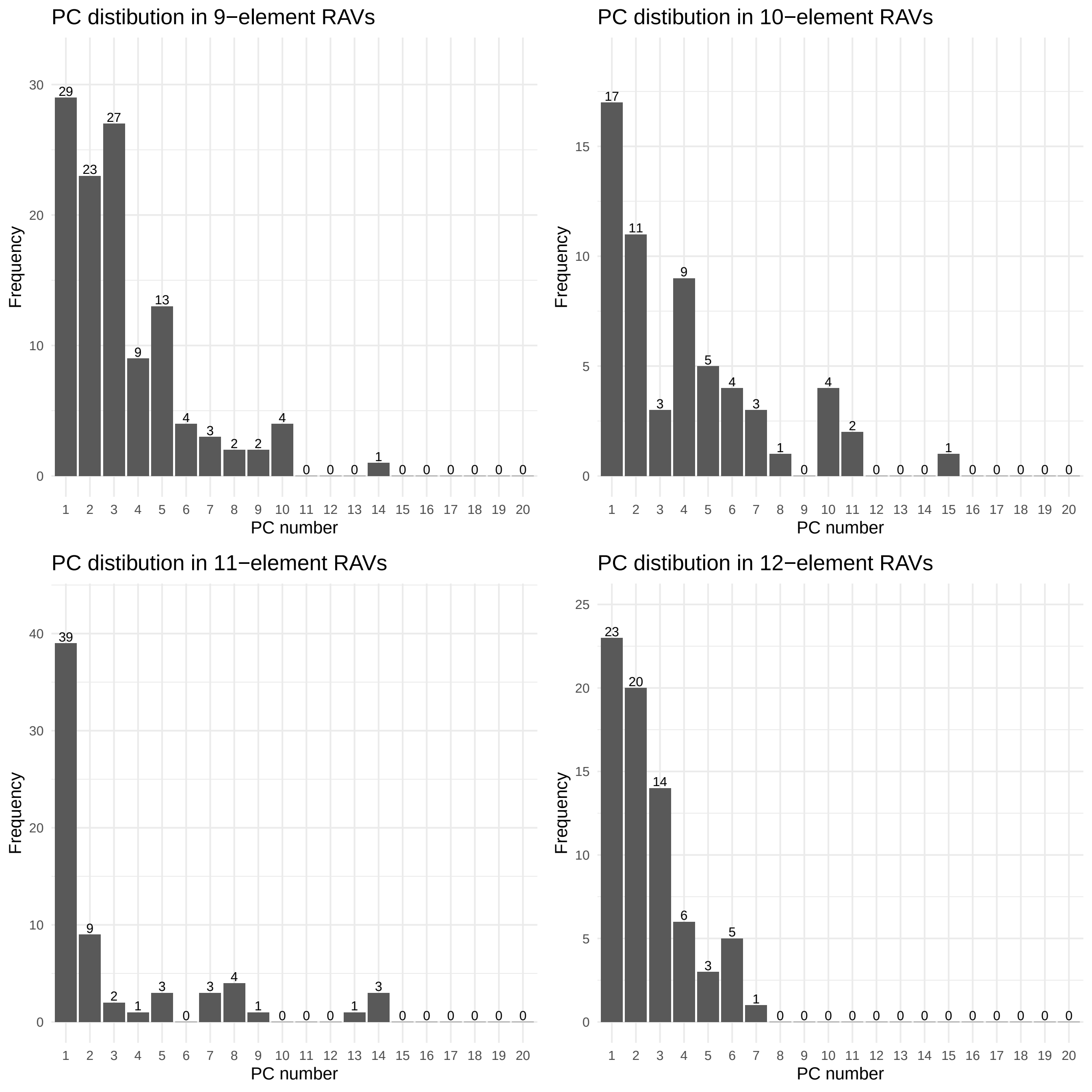
PC distibution in 9−element RAVs
Frequency
PC number
29 23 27 9 13 4 3 2 2 4 0 0 0 1 0 0 0 0 0 0
PC distibution in 10−element RAVs
Frequency
PC number
17 11 3 9 5 4 3 1 0 4 2 0 0 0 1 0 0 0 0 0
PC distibution in 11−element RAVs
Frequency
PC number
39 9 2 1 3 0 3 4 1 0 0 0 1 3 0 0 0 0 0 0
PC distibution in 12−element RAVs
Frequency
PC number
23 20 14 6 3 5 1 0 0 0 0 0 0 0 0 0 0 0 0 0

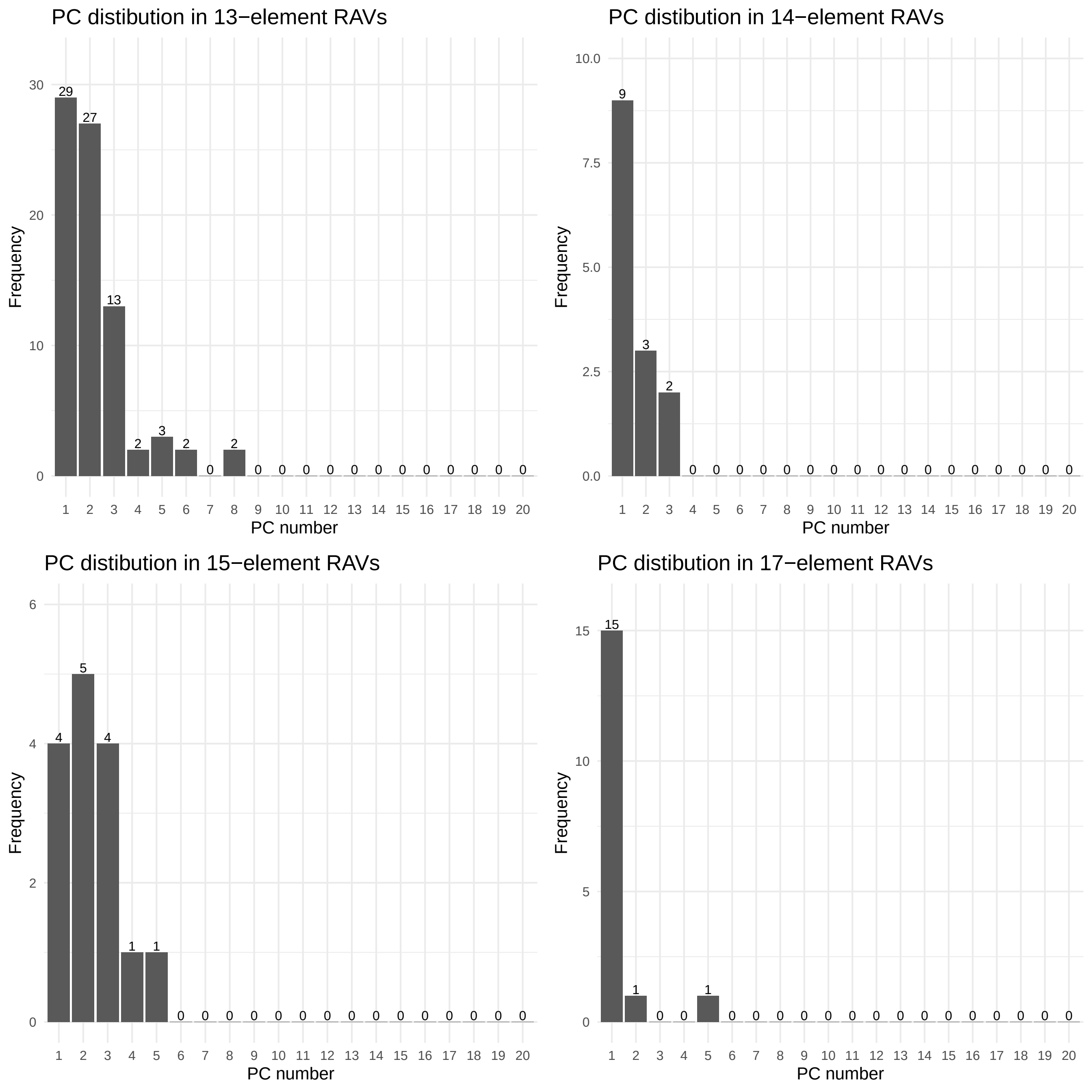
PC distibution in 13−element RAVs
Frequency
PC number
29 27 13 2 3 2 0 2 0 0 0 0 0 0 0 0 0 0 0 0
PC distibution in 14−element RAVs
Frequency
PC number
9 3 2 0 0 0 0 0 0 0 0 0 0 0 0 0 0 0 0 0
PC distibution in 15−element RAVs
Frequency
PC number
4 5 4 1 1 0 0 0 0 0 0 0 0 0 0 0 0 0 0 0
PC distibution in 17−element RAVs
Frequency
PC number
15 1 0 0 1 0 0 0 0 0 0 0 0 0 0 0 0 0 0 0

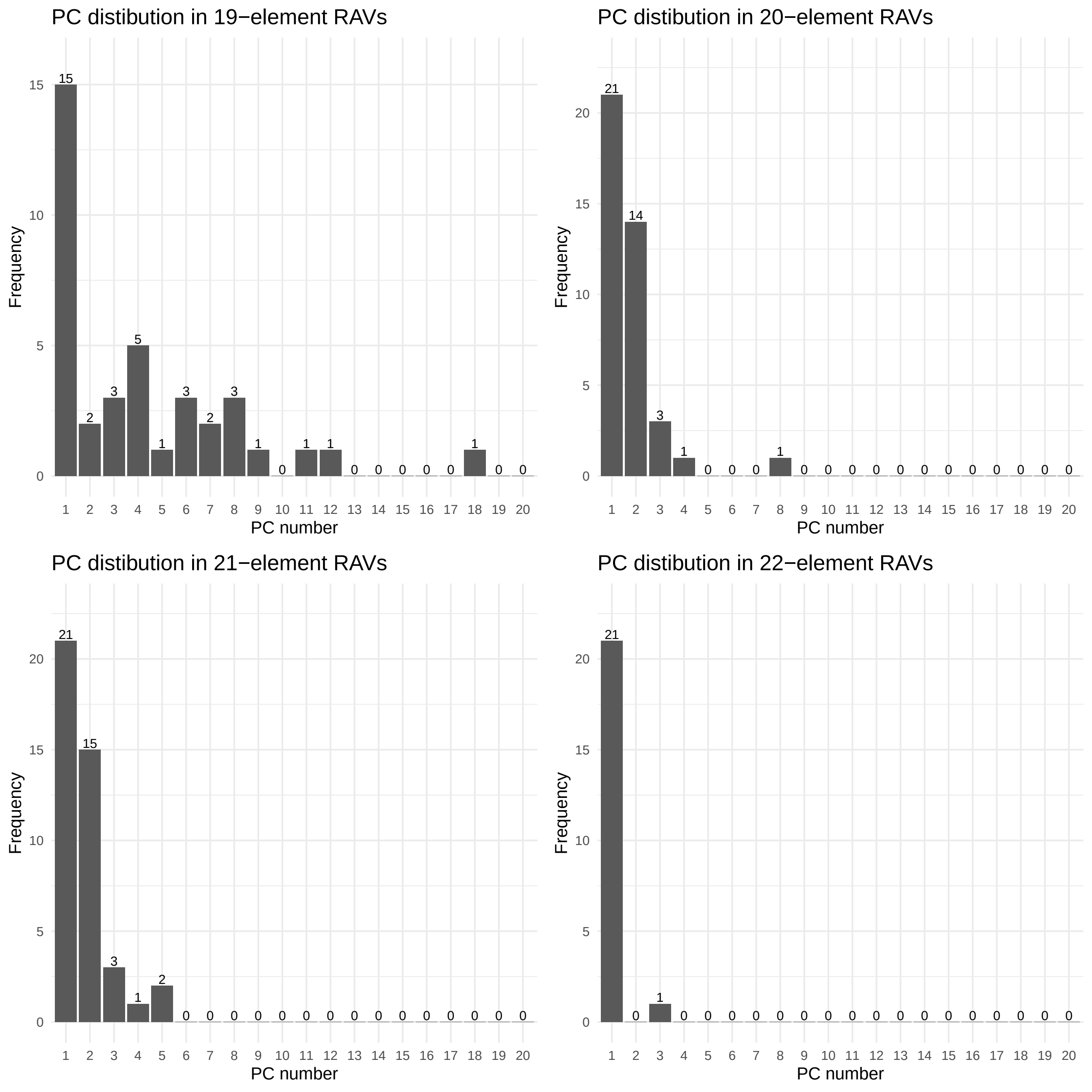
PC distibution in 19−element RAVs
15 2 3 5 1 3 2 3 1 0 1 1 0 0 0 0 0 1 0 0
Frequency
PC number
PC distibution in 20−element RAVs
21 14 3 1 0 0 0 1 0 0 0 0 0 0 0 0 0 0 0 0
Frequency
PC number
PC distibution in 21−element RAVs
21 15 3 1 2 0 0 0 0 0 0 0 0 0 0 0 0 0 0 0
Frequency
PC number
PC distibution in 22−element RAVs
21 0 1 0 0 0 0 0 0 0 0 0 0 0 0 0 0 0 0 0
Frequency
PC number

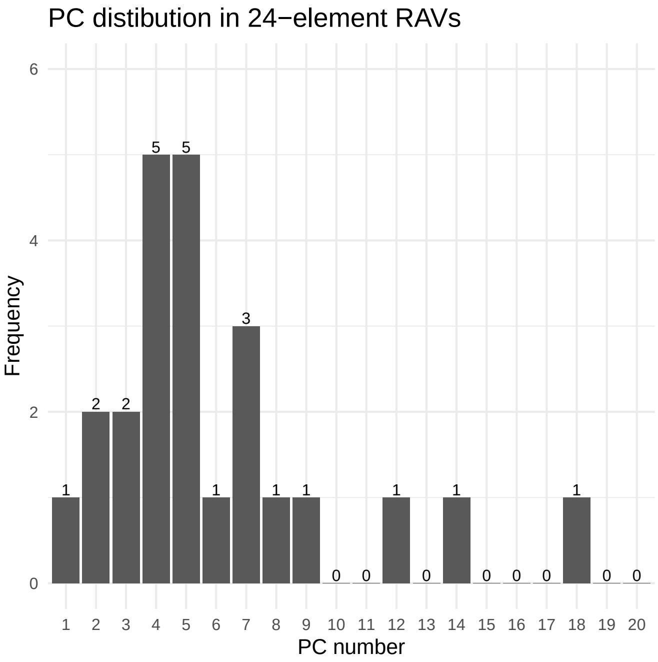

PC distibution in 24−element RAVs
Frequency
PC number
0
2
4
6
1 2 3 4 5 6 7 8 9 10 11 12 13 14 15 16 17 18 19 20
1 2 2 5 5 1 3 1 1 0 0 1 0 1 0 0 0 1 0 0
